# Prolonged Treatment with DNMT Inhibitors Induces Distinct Effects in Promoters and Gene-Bodies

**DOI:** 10.1371/journal.pone.0071099

**Published:** 2013-08-06

**Authors:** Yan-Fung Wong, Lars Martin Jakt, Shin-Ichi Nishikawa

**Affiliations:** Laboratory for Stem Cell Biology, RIKEN Center for Developmental Biology, Kobe, Japan; Florida International University, United States of America

## Abstract

Treatment with the demethylating drugs 5-azacytidine (AZA) and decitabine (DAC) is now recognised as an effective therapy for patients with Myelodysplastic Syndromes (MDS), a range of disorders arising in clones of hematopoietic progenitor cells. A variety of cell models have been used to study the effect of these drugs on the methylation of promoter regions of tumour suppressor genes, with recent efforts focusing on the ability of these drugs to inhibit DNA methylation at low doses. However, it is still not clear how nano-molar drug treatment exerts its effects on the methylome. In this study, we have characterised changes in DNA methylation caused by prolonged low-dose treatment in a leukemic cell model (SKM-1), and present a genome-wide analysis of the effects of AZA and DAC. At nano-molar dosages, a one-month continuous treatment halved the total number of hypermethylated probes in leukemic cells and our analysis identified 803 candidate regions with significant demethylation after treatment. Demethylated regions were enriched in promoter sequences whereas gene-body CGIs were more resistant to the demethylation process. CGI methylation in promoters was strongly correlated with gene expression but this correlation was lost after treatment. Our results indicate that CGI demethylation occurs preferentially at promoters, but that it is not generally sufficient to modify expression patterns, and emphasises the roles of other means of maintaining cell state.

## Introduction

Epigenetic changes are increasingly recognised as a major characteristic of many human diseases [1]. CpG dinucleotides are relatively uncommon and have an asymmetrical distribution throughout the human genome. Almost all CpG dinucleotides are methylated, except those located in CpG islands (CGIs), which lack DNA methylation setting them apart from bulk genomic DNA [2–4]. Aberrant methylation of CGIs in or near the promoter region of tumour suppressor genes (TSG) represents one of the most consistent hallmarks of human cancers [5] and these TSGs are often silenced in haematopoietic malignancies [6]. Thus, CGI methylation represents an ideal candidate for diagnostic and prognostic cancer markers [7].

Myelodysplastic syndromes (MDS) comprise a heterogeneous group of bone marrow disorders affecting mainly elderly patients [8]. A number of gene mutations and cytogenetic changes have been implicated in the pathogenesis of MDS, including mutations of RAS, TP53 and RUNX1, and more recently ASXL1, c-CBL, DNMT3A, IDH1/2, TET2, and EZH2 [9]. Nevertheless, these genetic abnormalities do not fully explain the pathogenesis of MDS because they are also commonly found in other myeloid malignancies and roughly 20% of MDS patients have no known genetic mutation [10]. On the other hand, hypermethylation of specific genes, such as p15, E-cadherin, ER, MYOD1, and HIC1, have been noted [11], and whole genome studies have revealed that MDS patients contain aberrant DNA methylation in thousands of genes compared to normal haematopoietic progenitor cells (HPC) [12].

The process of cytosine methylation is reversible and may be altered by biochemical and biological manipulation, making it an attractive target for therapeutic intervention [13]. Epigenetic therapy with hypomethylating drugs is now the standard of care for MDS [14]. Two prominent examples are the cytosine analogs 5-azacytidine (AZA) and 2’-deoxy-5-azacytidine (DAC). These are potent inhibitors of DNA methyltransferases (DNMTs) and have been approved for MDS treatment [15,16]. Recent efforts have focused on lowering the dosage of azacytidine and decitabine to reduce toxicity. However, the effect of low-dose treatment on the MDS methylome is still unclear. In this report, we have determined concentrations of AZA and DAC that allow prolonged treatment in a leukemic cell model (SKM-1), and have determined how this affects global CGI methylation using a microarray approach. Our results show that the methylome was selectively demethylated by low-dose treatments and that gene-body CGIs were more resistant to this process. We also provide evidence that prolonged low-dose AZA and DAC treatment is sustainably effective in modifying the epigenome.

## Materials and Methods

### Cell Culture and Reagent

SKM-1 (Japanese Collection of Research Bioresources Cell Bank, JCRB0118) cells were cultured in RPMI-1640 (Gibco) medium containing 10% fetal bovine serum (JRH Biosciences), 100 U/mL penicillin and 100 µg/mL streptomycin (Meiji). All cells were culture in an environment of saturated humidity, 5% CO2, and 37° C. Stock solutions (1mM) of 5-azacytidine AZA and 5-Aza-2’-deoxycytidine DAC (Wako chemicals) were stored at -80° C and diluted in tissue culture medium to the required concentrations before being added to cells in the exponential phase of growth.

### Cell Proliferation assay

SKM-1 cells were seeded into 6-well plates at 1x10^5^ cells, 24 hours before drug treatments. Cells were treated daily with AZA and DAC at final concentrations of 0nM (mock-treated control), 0.1nM, 1nM, 10nM, 100nM, and 1µM for 7 days with medium changes every 24 hours. Three hours before plate reading, 20µl of CellTiter 96 AQueous One Solution Cell Proliferation Assay (MTS) (Promega) was added into a 96-well plate containing 100µl of the treated samples. Cell proliferation was measured by OD 490nm and the relative cell proliferations were normalised by mock-treated control.

### Prolonged AZA/DAC treatment and western blotting analysis

Stocked AZA and DAC were added daily to cultures of SKM-1 cells for 4 independent experiments performed at two separate times. For sets 1 and 2, 2x10^5^ cells were cultured in 6-well plates with medium changed every 24 hours. For sets 3 and 4, 1x10^6^ cells were cultured in 90mm Petri dish plates with medium changed every 48 hours. Treatments were stopped at day 28 and the cells were grown in the absence of drugs for 10 days. Samples were collected on days 7, 14, 21, 28 and 38 for both genomic DNA and total mRNA purifications (DNeasy Blood & Tissue Kit and RNeasy Mini Kit, QIAGEN).

Protein extracts were also obtained from sets 3 and 4 after 28 days of treatment. Cells were lysed in RIPA buffer containing Halt Protease and Phosphatase Inhibitor Single-Use Cocktail (Thermo Scientific) for 5 minutes on ice. Whole-cell lysates were prepared in denaturing SDS sample buffer and subjected to 8% SDS-PAGE. Proteins were transferred to Immobilon-P membrane (Millipore, Billerica MA) and the blots were blocked with 5% non-fat dry milk in TBS-T buffer (50 mmol/L Tris-HCl, 200 mmol/L NaCl, 5% Tween 20). We used the following primary antibodies: anti-beta Actin (ab8226, 1:5000 dilution, abcam) as loading control, anti-Dnmt1 (ab13537, 1:1000 dilution, abcam), anti-Dnmt3a (ab13888, 1:1000 dilution, abcam), and anti-Dnmt3b (ab13604, 1:1000 dilution, abcam). Horseradish peroxidase conjugated secondary antibodies (NA931V, 1:10000 dilution, GE Healthcare Life Sciences) were detected using ECL prime western blotting detection reagent (GE Healthcare) and the Light-Capture system (ATTO).

### Methylcytosine fractionation assay

Global DNA methylation was assayed essentially as described [17] by comparing microarray signals from fragmented DNA with or without additional digestion with McrBC, an enzyme which cuts DNA between pairs of methylated CpG dinucleotides separated by 40-3000 bases of intervening DNA. Briefly, one microgram of intact genomic DNA from each sample was sonicated to generate DNA fragments sized between 8 and 10kb. The fragmented DNA was then optionally digested with 50 units of McrBC (NEB) for 16 hours at 37 degrees. Gel electrophoresis was used to select fragments ranging from 1 to 3kb and whole-genome amplification PCR (GenomePlex^®^ Complete Whole Genome Amplification Kit, SIGMA) was performed for 14 cycles with 20ng of eluted DNA samples.

### CpG-islands array analysis

DNA labelling and hybridisation was performed according to the supplied protocol (Agilent Microarray Analysis of Methylated DNA Immunoprecipitation Version 1.0). For each cell sample, 2.5 µg of McrBC- and mock-digested DNA were labelled with Cy5 and Cy3 respectively. Equal amounts of labelled samples were mixed and applied to Human CpG Island Microarrays (G4492A, Agilent). Methylation levels were estimated from the log (base 2) of the ratio of the intensity of signal from the undigested to digested DNA. Data was analysed by Agilent Genomic Workbench 5.0 and statistical analyses were performed using Bioconductor [18] and custom R code.

### Bisulfite sequencing and pyrosequencing

DNA (1µg) from each sample was bisulfite converted using sodium bisulfite (EpiTect Bisulfite kits, Qiagen) following the conditions suggested by the manufacturers. Gene-primers for amplifying 15 regions from the HOXA cluster, GFI1, KCNC4, RALA, KAZALD1, CCND1, TGIF2, ZNF800, SIRT1, GTF2F1, TYROBP, IL8, and TNF were developed using MethPrimer [19]. PCR was performed using EpiTaq HS enzyme (Takara Bio), according to the manufacturer’s instructions. The PCR products were gel-purified (QIAquick Gel Extraction Kit, Qiagen) and cloned into the TA vector (TOPO-TA cloning, Invitrogen). 10 clones from each sample were selected and the sequences were determined using an ABI PRISM® 3700 Genetic Analyzer (Applied Biosystems). Data was summarised by a web-based software; QUMA (http://quma.cdb.riken.jp/top/quma_main_j.html) [20].

LINE-1 element and GAPDH promoter methylation were estimated using the PyroMark LINE-1 and GAPDH CpG Assay (Qiagen). Primers for pyrosequencing HSPA2, TNF, and TYROBP were designed by PyroMark Assay Design 2.0 software (Qiagen). Pyrosequencing was conducted using a PyroMark Q24 instrument (Qiagen) and methylation levels were quantitated with the PyroMark Q24 1.010 software. Relative peak height differences were used to calculate the percentage of 5-methylcytosines at each given site. Percent methylation within a sample was subsequently determined by averaging across all interrogated CpG sites in the analysis. All primer sequences for bisulfite sequencing are shown in Table S1 in File S1.

### Gene expression analysis

One microgram of total mRNA was used in cDNA synthesis with the RT^2^ First strand Kit (Qiagen) or ProtoScript® M-MuLV First Strand cDNA Synthesis Kit (NEB). Hematopoietic Stem Cells and Hematopoiesis PCR Array (PAHS-054A, Qiagen) was used for profiling expression of 84 genes. Quantitative real-time PCR (QPCR) was performed by SYBR Green Mastermix (Applied Biosystems) on an Applied Biosystems 7900 or 7500 Real Time PCR system. Relative gene expression was determined based on the threshold cycles (Ct) of the genes of interest and the internal reference gene GAPDH [21]. Primer sequences for HSPA2, TNF, and TYROBP are shown in table S3 in File S1. For expression array analysis, two micrograms of total RNA were used to prepare biotinylated RNA using the Affymetrix One Cycle Target Preparation Protocol driven by T7-linked oligo(dT) primers. Samples were hybridized overnight to Affymetrix HG U133 Plus 2.0 arrays, scanned and processed using GeneChip Operating Software. Statistical analyses were performed using Bioconductor and custom R code. The eXintegrator system was used to visualise expression data and for selection of probe sets by internal probe co-variance.

### Data Analysis

Gene coordinates and CpG island positions were based on the HG18 assembly and were obtained from data files provided by the Agilent Genomic Workbench 5.0 system. CpG island positions were re-defined as regions contiguously (with a max inter probe distance of 250 bp) covered by probes, and overlapping CpG islands as indicated by Agilent data files. The resulting CpG island, gene and probe coordinates were then used to define relationships between genes, CpG islands and probes using a series of Perl scripts (Table S2 in File S1). Probes were assigned to the nearest or overlapping CpG islands. CpG islands in turn, were defined as, 1) upstream: upstream of, but not overlapping with the transcriptional start site (TSS), 2) gene body: lying entirely within the transcribed region, 3) promoter: overlapping with the TSS and the 5' region, 4) downstream: 3' of the transcript, 6) transcriptional termination site (TTS): overlapping the transcribed region and the 3' region, 7) Complete: where the complete transcribed region was contained within a CpG island and 8) Distant: with a distance of more than 3000 bp from the nearest gene (Table S3 in File S1) (This numbering is based on a binary OR combination of: upstream 1, gene body 2, downstream 4 and distant 8). The resulting probe-gene mapping was used to define an Agilent probe to Affymetrix probe mapping. A single Affymetrix probe-set was selected for each gene based on the probe pair covariance estimated by the f-statistic (between sample variance / within probe-set variance) using the eXintegrator system (http://www.cdb.riken.jp/scb/documentation/), and these were mapped to gene ids as provided by Agilent using the DAVID online resource. A total of 13721 Affymetrix probe sets from the HGU133_Plus_2 were mapped to 183160 probes.

**Figure 1:**
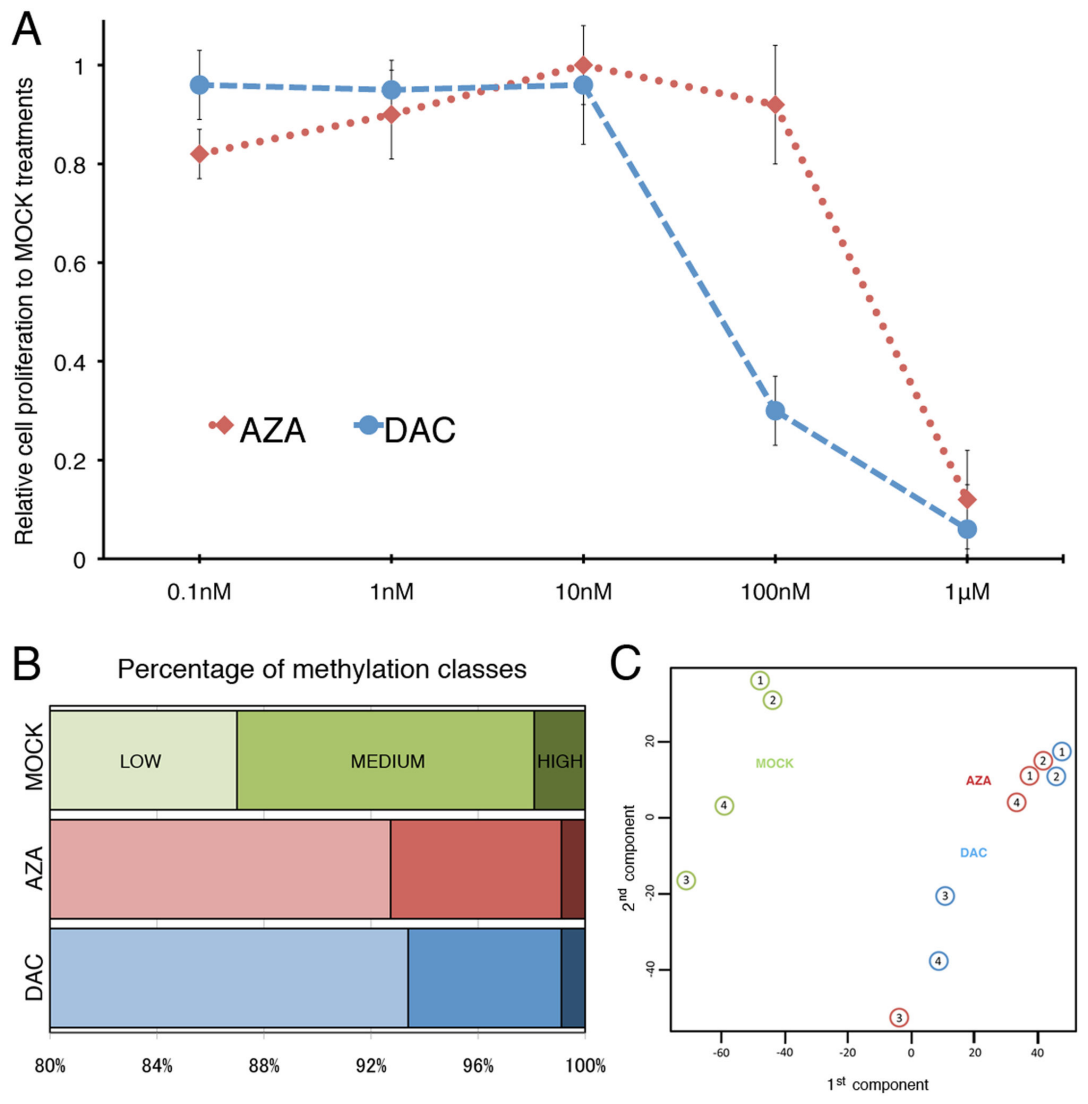
Prolonged treatments of AZA or DAC in SKM-1 cells. **A**, Cell proliferation was assayed using the CellTiter 96 Aqueous One Solution assay kit after treating SKM-1 cells for 7 days with different concentrations of AZA (red dotted line) or DAC (blue dashed line). The percentage of cell proliferation was calculated relative to the rate of proliferation in untreated cells, and obtained from the mean (± SEM) of three independent experiments. **B**, Probes targeting CGI regions were classified into hyper, intermediate or hypo methylation groups according to their log_2_-ratios obtained from microarray analysis (See materials and methods). Most probes were hypomethylated (>80% in all cases). The number of probes classified as intermediate or hypermethylated were reduced after AZA or DAC treatments. **C**, Principal Component Analysis (PCA) with data from 4 independent treatments (from 52915 probes selected by within-replicate variance).

**Figure 2:**
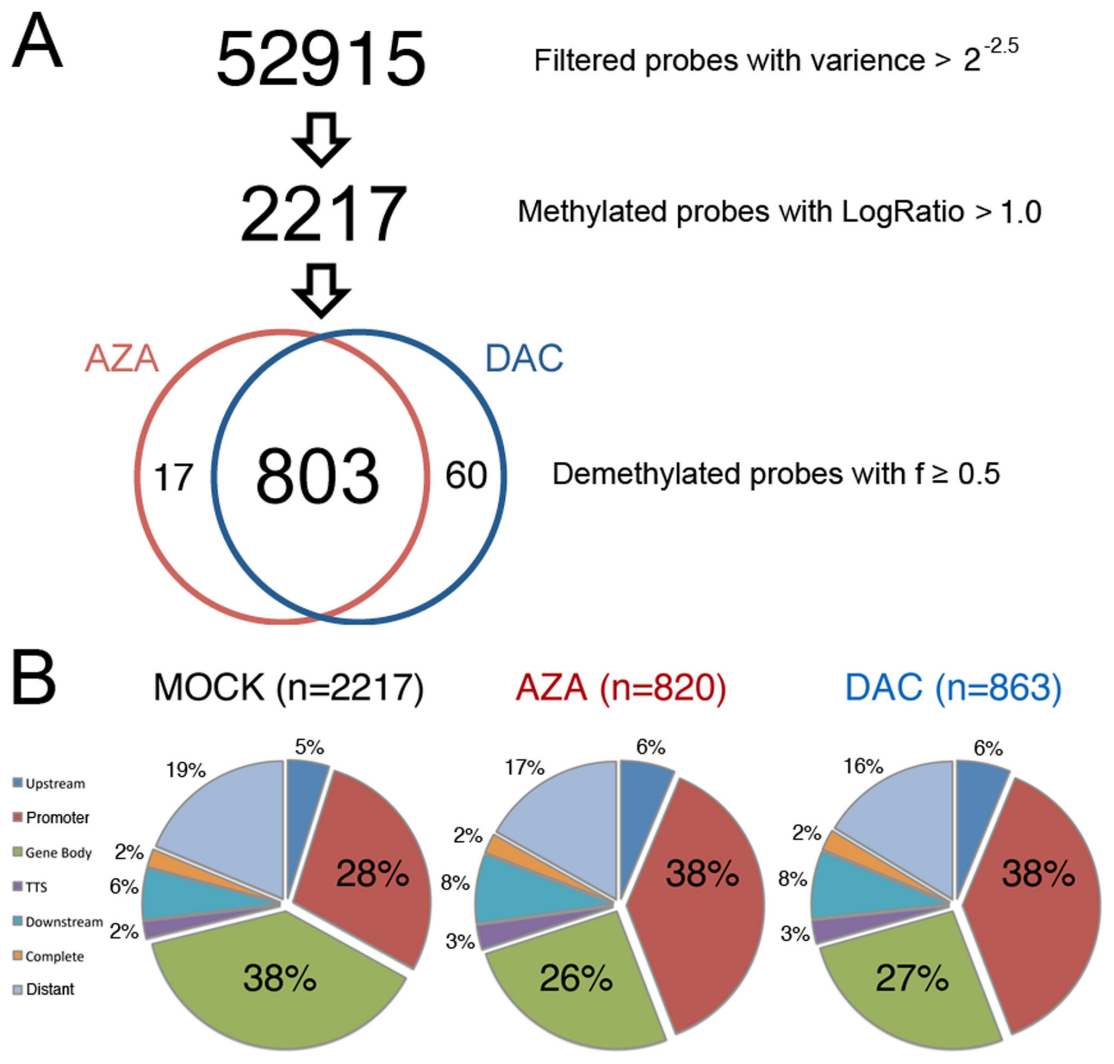
Selection of probes demethylated after AZA or DAC treatments. **A**, Schematic for classification of probes. Of 52,915 probes with low within-replicate variance, 2217 probes had mean log_2_-ratios larger than one in the control samples indicating more than 50% CG methylation. Probes whose between group variance was at least 0.5 times that of the sum of within group variances and whose log_2_-ratios was less than 1.0 after treatment were considered as demethylated. **B**, Distribution of gene feature classes within demethylated CGI probes. In mock-treated cells, the percentage of methylated probes in each class (log_2_-ratios higher than 1.0, 2217 probes) is shown. Demethylated probes were over-represented in promoters (p<2E-14) and under-represented in gene bodies (p<2E-20) in both AZA and DAC treated cells as determined by Fisher’s exact test and considering demethylated probes as being sampled from the control hyper-methylated set of probes.

The analysis presented for figures 1 and 2 was based on a subset of probes selected by their maximum variance within replicate groups (mock, AZA and DAC treatments 4 samples each). The threshold variance, 2^-2.5^, was chosen on the basis of a comparison of the distribution of variances within the replicate and mock replicate groups derived by an arbitrary permutation (Figure S1 and Table S4 in File S1), as well as by a manual inspection of log_2_-ratio values. This selected 52915 out of a total of 198302 probes. To identify probes that were demethylated as a result of the treatment we first identified probes from this selection (52915) that were methylated in the control (mock) samples (mean log-ratio > 1.0). De-methylated probes were then identified as probes from this subset that scored higher than 0.5 for a simplified f-statistic (between group variance / sum of within-group variance) and had mean log_2_-ratios below 1 in the treated sample. Over or under-representation of different classes (upstream, promoter, gene body, etc.) of CpG islands was tested individually by calculating the probability of the observed overlaps between the island classes and the demethylated probes using Fisher’s exact test as implemented by the R phyper function. Since we tested for both over and under-representation in the 7 different island classes we used a conservative p-value threshold of 0.001 (0.05/14 = 0.0034, but since the tests cannot be considered as independent we reduced this to 0.001, For the full set of p-values see Table S5 in File S1)**.**

**Figure 3:**
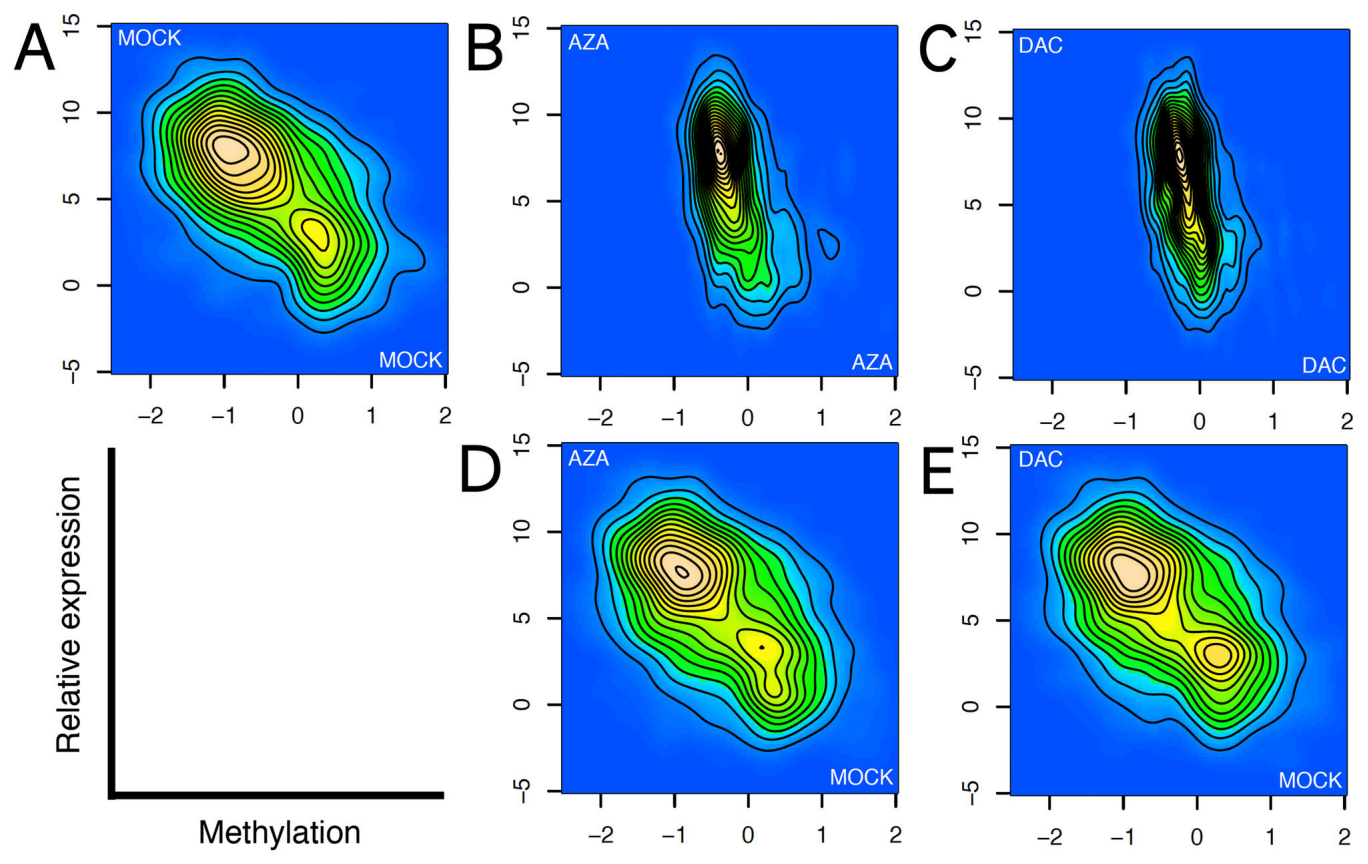
Correlation of gene expression and promoter CGI methylation. **A**) MOCK-MOCK (**B**), AZA-AZA (**C**), DAC-DAC (**D**), AZA-MOCK (**E**), and DAC-MOCK. Two-dimensional kernel density estimates of expression level (log_2_ transformed Mas5 estimates) and methylation levels (mean log_2_-ratios for the complete island) for a set of low-density CGIs associated with transcriptional start regions (from a total of 198302 probes). Methylation is on the x-axis, expression level on the y-axis. Top row shows plots for methylation plotted against expression levels within individual samples, bottom row shows expression levels in two treated samples plotted against methylation levels obtained from a single control sample as indicated. All plots are from a single representative sample.

Affymetrix expression values used in Figure 3, and Figure S6 and Figure S7 in File S1were obtained using the Bioconductor [22] implementation of the Mas5 method. Plots of expression vs. methylation used the kde2d function of the MASS package [23] Genes that were up or down-regulated as a result of AZA or DAC treatment were identified by a profile similarity search (sorted by mean Euclidean distance to a specified profile) implemented in eXintegrator, combined with a minimal two-fold change in mean expression values between treatment and mock samples.

## Results

### Prolonged low-dose AZA and DAC treatments induce DNA demethylation

Our experiment was designed to detect specific epigenetic alterations in leukemic cells after low-dose exposure to demethylating agents. We employed a monocytic leukaemia cell line, SKM-1, derived from a leukaemia patient with a historical background of MDS without chromosome 5q deletion [24] for low-dose treatments. It has been well documented that high-dose AZA or DAC treatment of several human cell lines induces DNA demethylation and gene re-activation within a day [25,26]. However, it also greatly reduces cell proliferation, viability, and induces apoptosis and is unlikely to mimic the effect of clinical therapy. To find conditions that may give rise to effects similar to those induced by drug treatment in patients, we set out to determine a treatment plan using dosages compatible with normal proliferation of SKM-1 cells over an extended period of time. Graded concentrations (from 0.1nM to 1mM) of the agents were applied daily to SKM-1 cells in fresh medium. After continuous treatment for a week, cell proliferation ceased at the micro-molar scale but remained constant at lower dosage (Figure 1A). SKM-1 cells were approximately 10 times more sensitive to DAC than AZA but otherwise appeared to respond in a similar manner.

We assigned the optimum concentrations of AZA and DAC for daily treatment of SKM-1 cells as 100 and 10 nM respectively. Four weeks of daily treatment showed no effect on SKM-1 cellular morphology, but was sufficient to reduce the amount of DNMT1 and DNMT3B proteins significantly (Figure S2 in File S1). Both AZA and DAC induced DNA demethylation at LINE-1 repetitive sequences, though neither could reduce the basal methylation level of the GAPDH promoter (Figure S3 in File S1).

We also measured the expression of a number of hematopoietic markers using PCR arrays. Both AZA and DAC treatment altered the expression of approximately half of these markers (such as CD4 and CD34) (Figure S4A and Figure S4B in File S1) and their effects were highly similar (Figure S4C in File S1). In contrast to prolonged low-dosage treatments, a short exposure (3 days) of micro-molar AZA (10mM) and DAC (1mM) resulted mainly in gene down-regulation (Figure S4D and Figure S4E in File S1) and greatly suppressed cell proliferation (Figure 1A). These observations indicate that this (low-dosage) treatment plan has the potential to mimic the standard clinical treatment for MDS patients.

### Demethylation pattern is regional and gene-specific

Changes in CGI methylation in tumours has been hypothesized to constitute a distinct phenotype (“CpG island methylator phenotype” or CIMP) [27,28] and demethylation of tumour suppressor CGIs was reported in MDS patients after treatments with AZA or DAC [12,25,29,30]. In order to analyse DNA methylation throughout the genome, we employed a method based on the ability of the McrBC enzyme to cut DNA at methylated CpG positions separated by 40 to 3000 bases. Digestion of fragmented DNA with this enzyme followed by comparative hybridisation to micro-arrays with non-digested DNA has been shown to be an effective means of detecting differentially methylated regions (DMR) [17]. Following McrBC digestion labelled probes were hybridized on microarrays containing probes tiled across all CGIs (See materials and methods). To correlate DNA methylation with microarray data, we used bisulfite sequencing to quantify DNA methylation at a selection of CGI probes lying in the HOXA cluster. A fifty percent level of methylation corresponded approximately to a log_2_ ratio (log_2_-ratios of signal from undigested to McrBC digested DNA) of 1 (Figure S5A in File S1).

To reduce the influence of noise from badly performing probes we first selected a set of 52915 probes (from a total of 198302) that showed low within replicate variance. These probes were then classified into methylation high (log_2_-ratios > 1.5), low (log_2_-ratios<0.5), and medium (log_2_-ratios from 0.5 to 1.5) classes. Most (86.9%) CGIs fell into the methylation low category, and only a small fraction of regions were highly methylated (1.9%) (Figure 1B).

We mapped CpG islands according to their overlap or proximity to gene features (see material and methods). The percentage of highly methylated probes around promoters was significantly under-represented (p<6E-66) whereas probes associated with the gene bodies were over-represented (p<1E-20) (Figure S5B in File S1). Next, we aimed to identify probes demethylated as a result of treatment in all experiments (two sets of experiments using slightly different culture conditions, see materials and methods). Prolonged AZA and DAC treatment reduced the number of methylation high and medium probes by half (Figure 1B) (with DAC showing a slightly stronger effect for the medium group). Principal component analysis (PCA) indicated that AZA and DAC treatments had a global effect on CGI methylation with treated samples clustered away from those in mock-treated controls (Figure 1C).

Since DNMT inhibition results in a decrease in methylation across the genome it is possible that this may affect the accuracy of array-based estimates of methylation through implicit or explicit normalisation procedures. A comparison of log_2_-ratios for treated and control samples indicated that a reduction in methylation occurs at the vast majority of methylated regions (Figure S6A and Figure S6B in File S1) and that AZA and DAC have very similar effects (Figure S6C in File S1). However, probes with low log_2_-ratios in the control samples generally showed higher log_2_-ratios in treated samples. To determine the cause of this, we performed bisulfite sequencing for all 12 samples for regions showing an increase, and ones showing a decrease in methylation after treatment (Figure S7 in File S1). This indicated that increases in log_2_-ratios after treatment at regions hypomethylated in control samples do not represent increases in methylation, and are likely caused by inappropriate normalization. More pleasingly however, this analysis shows a strong linear relationship between percent methylation and log_2_-ratios for regions with more than 10% of methylation. Importantly this relationship is identical across all samples thus validating our primary data.

From the selected 52915 probes, only 2217 CGI probes had log_2_-ratios higher than 1.0 (suggesting more than 50% methylation) in control samples (Figure 2A). Of these, a total of 880 and 803 probes were demethylated by at least one of, or both, drugs respectively (Table S6 in File S1). Probes representing promoter CGIs were over-represented (p<2E-14) whereas probes associated with gene bodies were under-represented (p<2E-20) (Figure 2B and Table S5 in File S1) in the identified sets. In summary, our result shows that low-dose AZA and DAC treatment can effectively induce CGI demethylation at promoters, while methylation is maintained within gene bodies.

### AZA and DAC treatments result in sustained gene activation

We next examined the correlation between expression and methylation levels. We performed transcriptome analyses for both mock and drug treated SKM-1 cells. The level of methylation in individual islands was summarised by the mean log_2_-ratios, and these were plotted against expression levels. Since individual genes can overlap multiple CGIs we divided the CGIs into classes depending on their overlap with gene features as described above and made separate plots for each class (Figure S8 in File S1). In the control cells, a clear anti-correlation between gene expression and methylation was observed for CGIs overlapping promoter elements. This correlation was stronger for promoter CGIs with low CG content (Figure S9 in File S1), which may be due to the general paucity of highly methylated high CG density CGIs. The data also suggested that relationships between expression levels and DNA methylation exist at non-promoter CGIs (eg. gene body, upstream / downstream). However, these relationships were not as robust with observations depending on the summary statistics used (Figure S10 in File S1), and apparently restricted to subsets of islands within each class rather than generally true for the full set of islands.

Interestingly, this anti-correlation was lost or markedly reduced in AZA and DAC treated cells (Figure 3B, Figure 3C **,** and Figure S11 and Figure S12 in File S1). However, expression levels within AZA and DAC treated cells were still anti-correlated against promoter methylation levels in control cells (Figure 3D, Figure 3E). This strongly suggests that promoter CGI demethylation was not generally sufficient to modify expression patterns, and emphasizes the roles of other means of maintaining cell state.

Although a correlation between CGI demethylation and up-regulation of gene expression was not generally observed, we identified a small number of genes where expression appeared to change following demethylation (Table 1). It should be emphasised that more than half (110/190) of the genes whose expression was more than two times higher in AZA and DAC treated samples (Table S**7** in File S1) were not associated with CGIs, and no array based methylation data was obtained for these genes. However, DNA demethylation was detected in the non-CGI promoters of the top three up-regulated genes (TYROBP, IL8, and TNF) by bisulfite sequencing (Figure S13 in File S1). These data indicate that prolonged low dose treatments are capable of demethylating CpG sites at non-CGI promoters and that this may have an effect on gene expression.

**Table 1:** Summary of AZA and DAC regulated genes following CGI demethylation.

				AZA (100nM)		DAC (10nM)		CD34+ (CB)	
Gene name	Genomic position	Genomic feature	CpG no.	Methylation	Expression	Methylation	Expression	Methylation	Expression
PFN2	Chr3:151170547-151170593	TS region	177	0.8 (1.4)	3.2 (1.5)	0.3 (1.4)	3.4 (1.5)	-0.8	0.4
TFAP2A	Chr6:10512822-10512866	Gene body	92	0.1 (1.3)	7.0 (1.7)	0.1 (1.3)	5.6 (1.7)	-0.7	0.3
	Chr6:10512927-10512971	Gene body	92	0.5 (1.7)		0.5 (1.7)		-0.7	
	Chr6:10522087-10522146	Gene body	24*	-0.1 (1.3)		-0.1 (1.3)		-0.8	
FLOT1	Chr6:30820417-30820464	TS region	161	1.0 (1.4)	7.2 (2.7)	0.7 (1.4)	7.1 (2.7)	7.2	11.4
PKIB	Chr6:122972782-122972831	Gene body	61*	0.1 (2.1)	20.0 (6.0)	0.2 (2.1)	23.5 (6.0)	-1.1	1.4
KLF6	Chr10:3814080-3814124	Gene body	19*	0.5 (1.4)	2.2 (1.1)	0.4 (1.4)	2.4 (1.1)	1.7	0.7
KCTD12	Chr13:76357225-76357269	TS region	180*	-0.7 (1.2)	3.4 (1.5)	-0.7 (1.2)	5.5 (1.5)	-1.0	5.0
	Chr13:76357284-76357328	TS region	180	-0.5 (1.1)		-0.3 (1.1)		-0.8	
HSPA2	Chr14:64076531-64076575	TS region	205*	-0.3 (1.6)	2.8 (1.3)	-0.4 (1.6)	2.8 (1.3)	-1.2	0.7
FOS	Chr14:74816197-74816245	TS region	226	0.4 (2.1)	13.4 (5.3)	-0.1 (2.1)	15.9 (5.3)	-1.1	4.9
	Chr14:74816521-74816565	TS region	226	0.6 (1.2)		0.6 (1.2)		0.7	
GPX4	Chr19:1055617-1055661	TS region	229	0.7 (1.1)	39.0 (14.8)	0.5 (1.1)	35.7 (14.8)	1.5	34.1
NCAPH2	Chr22:49312596-49312640	Downstream	538	-0.7 (1.4)	3.9 (1.3)	-0.5 (1.4)	3.1 (1.3)	1.6	0.1
	Chr22:49312722-49312766	Downstream	538	-0.4 (1.7)		-0.2 (1.7)		1.1	
	Chr22:49312787-49312831	Downstream	538	-0.5 (2.9)		-0.2 (2.9)		2.0	

( ) shows methylation or expression value from mock-treated SKM-1 cells

* Indicates probes located outside CGI

**Figure 4:**
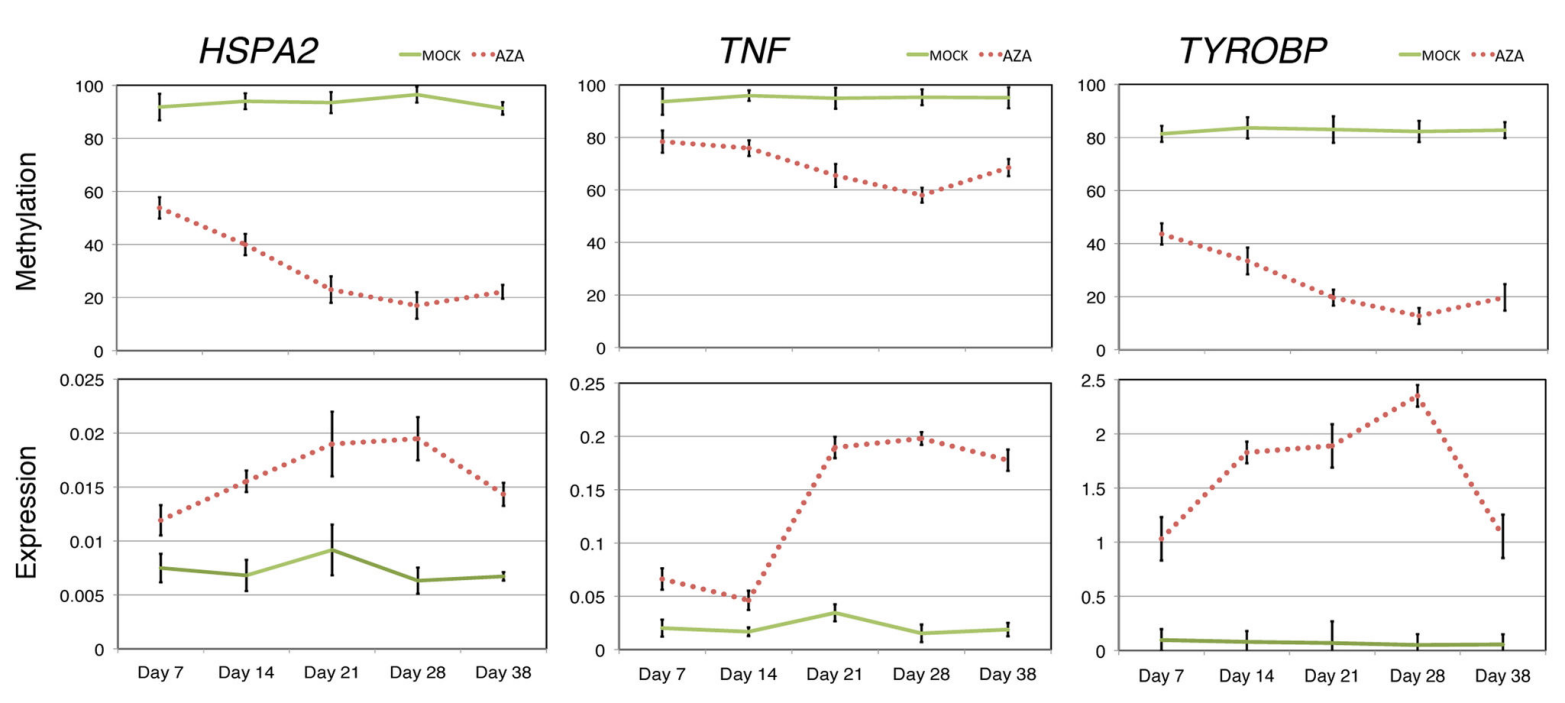
Time-course methylation and expression analysis for HSPA2, TNF, and TYROBP in AZA treated SKM-1 cells. **Top**, CG methylation as determined by pyrosequencing. Y-axes indicate mean percentage of methylation at 3 (HSPA2), 5 (TNF), and 4 CpG sites (TYROBP). **Bottom**, qRT-PCR data showing relative gene expression normalized to the housekeeping gene GAPDH. No drug was applied to the cells from day 28 to day 38. Both expression and methylation data are means (± SEM) from four independent experiments obtained from mock- (green line) and AZA- (red dashed line) treated cells.

We selected three candidates showing a ten-fold increase in expression after treatment; HSPA2, TNF, and TYROBP, to further characterize the action of the drug treatment. DNA methylation and expression profiles were determined for AZA-treated cells collected at days 7, 14, 21, and 28. Partial demethylation was detected by pyrosequencing at week one, and the methylation levels decreased gradually (Figure 4) throughout the treatment. Gene expression levels showed an inverse correlation to the methylation pattern throughout the assay. More importantly, these modifications were maintained in the absence of drugs for ten days.

## Discussion

The impact of demethylating agents on AML cell lines has recently been evaluated in several studies using bisulfite-modified target DNA arrays [31–33]. Here we have extended previous observations by investigating the effect of prolonged low-dosage treatment with AZA and DAC in a model, which is likely to be more similar to the clinical situation than previous short-term and/or high-dose treatments. Furthermore, we have investigated the effects in the SKM-1 cell line, which was derived from overt leukaemia following MDS and hence may provide a better model for investigating the relationship between demethylating treatments and MDS. We have used McrBC fragmentation in combination with standard CpG island arrays to robustly distinguish differential CGI methylation profiles in cells proliferating normally. Most of the CGIs are located at either TSS or within gene bodies (Table S3 in File S1). Gene-body CGIs are significantly more highly methylated than TSS CGIs. However, this epigenetic mark was preferentially lost at TSS CGIs after prolonged treatment with AZA or DAC.

Demethylating agents are thought to act as nucleoside analogues that incorporate into DNA, causing specific inactivation of DNMT1 [34,35]. This effect is non-specific and cannot per se explain the selectivity of demethylation observed. In contrast, the *de novo* methyltransferase DNMT3B are targeted to specific loci [36] and it is possible that their activity contributes to the specificity of the demethylation observed. However, we found a decrease in both DNMT1 and DNMT3B protein levels as a result of AZA or DAC treatment and hence it is unlikely that DNMT3B plays a strong role in the maintenance of DNA methylation at demethylation resistant loci (we were unable to detect DNMT3A protein, and mRNA levels were low in all samples). DNMT1 recognizes hemi-methylated DNA and causes the methylation of the non-methylated strand [37]. A reduction in the level of active DNMT1 should thus lead to the presence of more hemi-methylated DNA resulting in a passive demethylation during cell proliferation. It is not known whether DNMT1 is differentially targeted to different genomic regions in SKM-1 cells, but it is feasible that since DNMT1 recognizes hemi-methylated DNA, that it may be preferentially associated with regions of DNA containing high levels of methylated CpGs. In fact, genome-wide mapping data of DNMT family proteins suggests that DNMT1 is depleted in TSS and enriched in the gene bodies [36]. On the other hand, active DNA demethylation mediated by the TET family of methylcytosine deoxygenases [38,39] may also play a part in selectivity of demethylation. The Tet1 protein binds preferentially to TSSs and less intensively throughout gene bodies [40,41]. Therefore, a reduction in overall activity of DNMTs may have a stronger demethylation effect at regions that are normally less methylated, such as promoter regions.

A similar study on the effect of nanomolar-scale demethylating agents on both AML and breast cancer cell lines has recently been reported [33]. The authors of this study concluded that low-dose DAC affected a sub-population of clonogenic cells, rather than directly inducing cytotoxicity, to produce an antitumor “memory” response. These effects were accompanied by sustained promoter demethylation and gene re-expression in key cellular regulatory pathways. In agreement with their data, genes involved in the immune response of the 'Triggering Receptor Expressed on Myeloid Cells' (TREM)-1 signalling pathway (CCL3, FOS, IL1Β, JUN, IL8, TNF, and TYROBP) were activated after low-dose DNMT inhibitor treatment (Table S6 in File S1). Moreover, we demonstrate that the enhanced expression of a subset of these molecules (IL8, TNF, and TYROBP) follows DNA demethylation during the course of treatment. Activation of the TREM-1 signalling pathway is a feature of mature differentiated myelomonocytic cells [42]. TYROBP (also named as DAP12) constitutively associates with TREM-1 to mediate the induction of intracellular signals that lead to inflammatory cytokine TNF-a and chemokine IL-8 production [43]. Further investigation into the epigenetic regulation of the TREM-1 pathway may extend our knowledge of the molecular basis of hematopoiesis and myeloid cell differentiation.

The hypermethylation of CGIs located in promoter regions of tumour suppressor genes is now recognized as an important mechanism for gene inactivation [5]. However, demethylation of hypermethylated CGIs does not generally correlate with gene activation, as demonstrated here and elsewhere [26,44,45]. Recently, it has also been shown that only a minority of DAC-mediated demethylated promoters are associated with nucleosome remodelling [46]. Chromatin remodelling is required for gene reactivation after DNA demethylation as induced by DAC treatment [47] and the combination of DNMT and histone deacetylase (HDAC) inhibitors has been shown to induce re-expression of tumour suppressor genes in ovarian and colon cancer cell cultures [48,49]. A phase I study of DAC in combination with suberoylanilide hydroamic acid (SAHA) in patients with a range of tumour types has been reported [50]. We show here that CGI demethylation is not generally sufficient to change gene expression. However, it may change the epigenetic niche providing a permissive environment for histone remodelling. In this study, we have established an *in vitro* model of the epigenetic modification following prolonged treatment of demethylating agents. Since the effect was maintained after the cessation of treatment, it may provide a useful tool for testing the effects of histone modifying agents in a reduced DNA methylation environment.

The data-set provided with this work provides a rich resource for further analysis related to both DNA methylation in general, the effect of demethylating agents at pharmacological dosages and to the epigenetic changes that underlie myelodysplastic syndrome. We believe that the full value of this can only be realised in combination with clinical data and we present it here as to make it available for further analysis.

## Supporting Information

Figure S1 Distribution of internal variances. The variance around the mean of within group replicates was calculated for each probe and replicate group (red: control, AZA and DAC) as well as for a set of equally sized permutated sample groupings (each sample group contains a mixture of different treatment samples). The plot shows the distribution of the variances for each group. The vertical line indicates the 2^-2.5^ threshold used to select probes.
(TIF)

Figure S2 AZA and DAC reduced DNMT1 and DNMT3B proteins. Western blot analysis of DNMT1 and DNMT3B was performed with whole cell extracts from SKM-1 cells treated with either AZA (100nM) or DAC (10nM) continuously for 28 days. ACTB was used as loading control. Only set-3 and 4 treatments were subjected to the analysis.
(TIF)

Figure S3 Effect of prolonged AZA and DAC treatments on DNA methylation. **A-B**, Pyrosequencing of LINE1 and GAPDH for mock- and drug-treated cells. Percentage of methylation was calculated as the mean (± SEM) at 4 (LINE1) and 5 CpG sites (GAPDH) in four independent experiments.
(TIF)

Figure S4 Effect of DAC and AZA treatment on expression of 84 Hematopoietic markers. **A-C**, low-dose (100nM AZA, 10nM DAC) treatment for 28 days. **D–F,** high-dose (10mM AZA, 1mM DAC) treatment for 3 days. Relative expression is shown as M-Ct where M was defined as 3 more than the maximum Ct value obtained. Ct values for undetected genes were set to M to allow visualisation.
(TIF)

Figure S5 CGI methylation quantification and distribution of hypermethylated probes. **A**, log_2_-ratios from microarray data were plotted against average methylation value from bisulfite sequencing at 15 genomic regions in the HOXA cluster. The trend line intersects 50% of methylation at log_2_-ratios of 1.0. B, Percentages of probes (from a subset of 52195 probes) from different island classes as defined by their location with respect to gene features (left). Hypermethylated probes (log_2_-ratios > 1.5) were under-represented in promoters (p<6E-66) and overrepresented in gene bodies (p<1E-20) (right).
(TIF)

Figure S6 AZA and DAC induced demethylation. Probe log_2_-ratios from AZA (A) and DAC (B) treated samples were plotted against MOCK treatment log_2_-ratios. (C) AZA and DAC log_2_-ratios plotted against each other indicating an equivalent effect.
(TIF)

Figure S7 Correlation between microarray log_2_-ratios values and bisulfite sequencing across treatment groups. Bisulfite sequencing was performed for all sample series for MOCK, AZA, and DAC treated samples for genomic regions showing either increases or decreases in methylation after treatment. The percentage of methylation determined by bisulfite sequencing was plotted against log_2_-ratios from microarray data. Log_2_-ratios show a clear correlation with percent methylation for regions with more than 10% of CG positions methylated, but show no clear correlation for lower levels of methylation.
(TIF)

Figure S8 Correlation of gene expression and CGI methylation for different genomic features. Expression in mock treated samples (log_2_ transformed Mas5 values) derived from Affymetrix arrays were plotted against CGI methylation (mean log_2_-ratios for probes from individual islands) for probes lying in CGIs associated with (A) Upstream, (B) promoter, (C) gene body, (D) TTS, (E) downstream, (F) complete, (G) distant regions (see materials and methods for definitions).
(TIF)

Figure S9 Figure S9. Correlation of gene expression and CGI methylation in promoters with different CpG densities. For mock-treated SKM-1 cells, expression (as in S8 & S9) was plotted against CGI methylation for all probes associated with promoter CGIs **(A)**, and for those associated with the 5th lowest **(B)** and highest **(C)** CG density promoter associated islands.
(TIF)

Figure S10 Mock treated cells; Correlation of expression and island methylation summary statistics for different CGI classes. Expression was plotted against several CGI methylation summary statistics for the different classes of CGI as in Figure S8. CGI methylation was summarised by the minimum, mean, median or maximum probe log-ratio. The use of the minimum log-ratio suggests a relationship between gene expression at non-promoter islands (upstream, gene body, downstream and complete), but this does not appear to be a general relationship and may be true for a subset of the probes only.
(TIF)

Figure S11 AZA treated cells; Correlation of expression and island methylation summary statistics for different CGI classes. Plots as in S10 for an AZA treated sample. The relationships between methylation and expression observed in control samples are no longer present.
(TIF)

Figure S12 DAC treated cells; Correlation of expression and island methylation summary statistics for different CGI classes. Plots as in S11 for a DAC treated sample.
(TIF)

Figure S13 Bisulfite sequencing analysis. The extent of CpG methylation was determined for target sequences within FOXD1, IL8, TNF, and TYROBP by bisulfite sequencing for DNA derived from mock and prolonged AZA treated SKM-1 cells. Bisulphite sequence data from 10 independent clones are shown. Black circles represent methylated CpG sites. Dots marked with a cross were not analyzable by sequencing.
(TIF)

Table S1 Primer sequences used in this study. (PDF)

Table S2 Relationship between genes, CpG islands, and probes. Columns are, 1) Internal gene-id; in the case of islands distant from genes this is set to -1, 2) Gene symbol, 3) strand, 4) Chromosome, 5) and 6) gene start and end respectively, 7) internal island id, 7) island CpG count, 8) and 10) CGI start and end, 11) distance of island from gene, 12) and 13) CGI-gene relationship code* with respect to nearest and second nearest gene, 14) internal probe id, 15) probe name, 16) and 17) probe start and end, 18) probe to CGI distance, 19) probe to gene distance, 20) and 21) probe-gene relationship codes for nearest and second nearest genes. *The CGI-gene relationship code refers to the respective positions of CGI and gene as outlined in the methods section (1: upstream, 2: gene body, 3: TSS, 4: downstream, 6: TTS, 7: complete, 8: distant). The probe relationship code is similar but for individual probes.
(XLSX)

Table S3 Numbers of probes lying within different classes of CGI. The numbers of probes from each class was calculated for all (top row) or for probes with a maximal log_2_ within-group variance below the indicated thresholds (as used to select probes for further analysis in the main section).
(PDF)

Table S4 Proportions of probes lying within different classes of CGI. The proportions of probes from each class were calculated for all (top row) or for probes with a maximal log_2_ within-group variance below the indicated thresholds. No change in proportion is seen as probes are selected by increasing stringency.
(PDF)

Table S5 P-values for over- and under-representation of CGI class representation in demethylated probes. Probes demethylated as a result of AZA or DAC treatment were determined as described in the main text. These were considered as having been sampled from the set of probes hyper-methylated in the control samples. The probability of observing at least or at most the number of probes identified from each individual class was calculated using Fisher’s exact test for over- and under-representation respectively.
(PDF

Table S6 CGI methylation data for 803 demethylated regions in mock and drug-treated SKM-1 cells. Columns as indicated. CpG no. refers to the number of CpG positions within the associated CGI. Values are given as the mean log-ratio of four replicate samples; variances indicate the maximum internal variance for the three replicate groups; fscore indicates the variance between sample means divided by the sum of the internal variances.
(XLS)

Table S7 Expression data with gene regulation > 2 folds for mock and drug-treated SKM-1 cells. SKM, AZA, and DAC refer to expression level of corresponding samples MOCK-, AZA-, and DAC-treated cells respectively. AZA/SKM is the ratio between AZA- and MOCK-treated SKM cells. DAC/SKM is the ratio between DAC- and MOCK-treated SKM cells. DEM/CTL is the mean of ratios from AZA/SKM and DAC/SKM.
(XLS)
