# Supplementary figures and images for: Prolonged Treatment with DNMT Inhibitors Induces Distinct Effects in Promoters and Gene-Bodies
Source: PLoS One. 2013 Aug 6;8(8):e71099. doi: 10.1371/journal.pone.0071099 (PMC3735498; doi:10.1371/journal.pone.0071099)

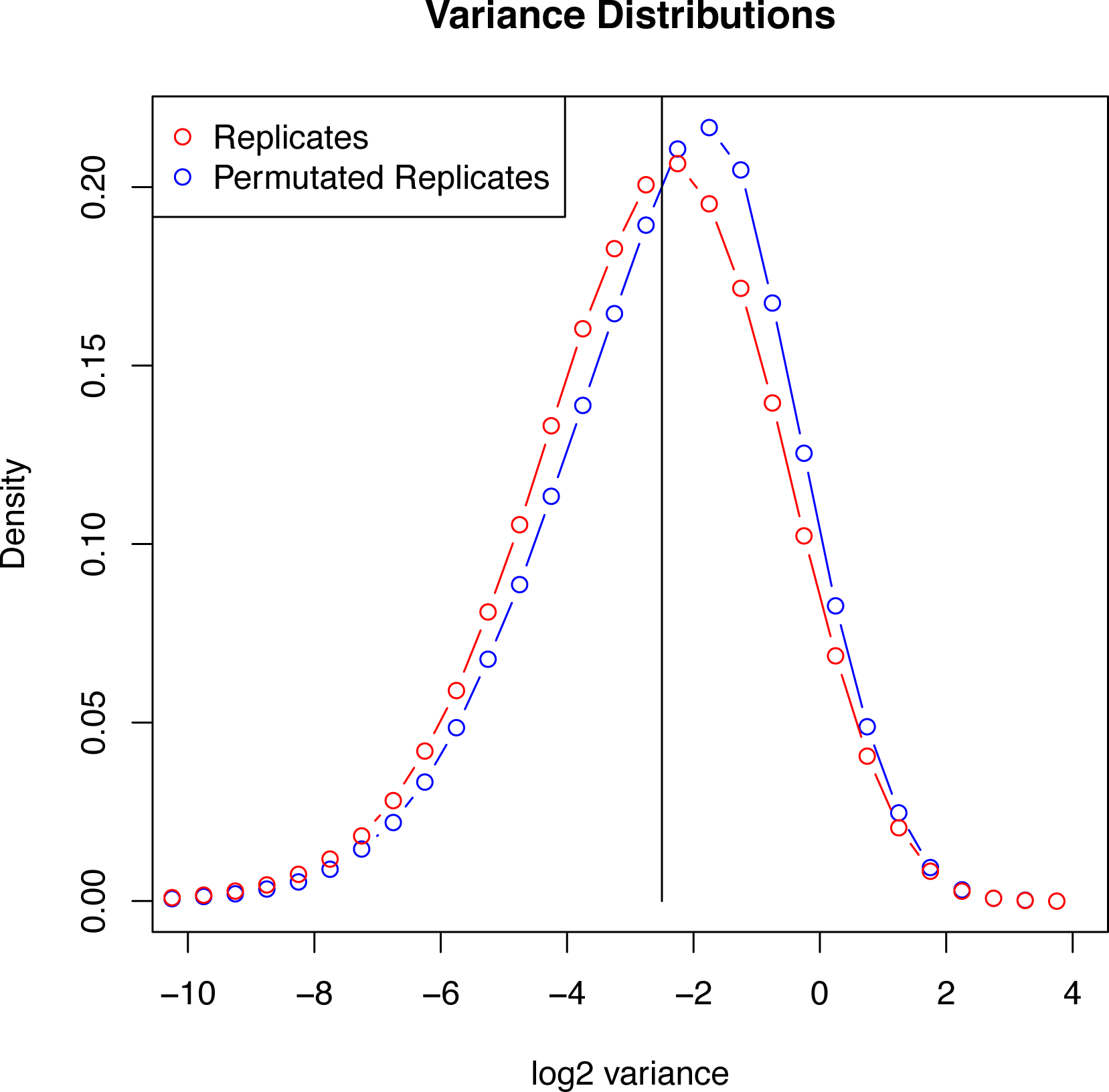

Supplement: Figure S1 — The variance around the mean of within group replicates was calculated for each probe and replicate group (red: control, AZA and DAC) as well as for a set of equally sized permutated sample groupings (each sample group contains a mixture of different treatment samples). The plot shows the distribution of the variances for each group. The vertical line indicates the 2-2.5 threshold used to select probes. (TIF) [file pone.0071099.s001.tif]

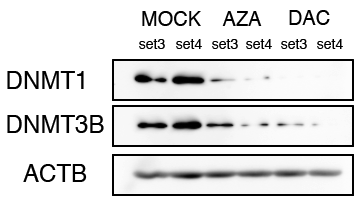

Supplement: Figure S2 — Western blot analysis of DNMT1 and DNMT3B was performed with whole cell extracts from SKM-1 cells treated with either AZA (100nM) or DAC (10nM) continuously for 28 days. ACTB was used as loading control. Only set-3 and 4 treatments were subjected to the analysis. (TIF) [file pone.0071099.s002.tif]

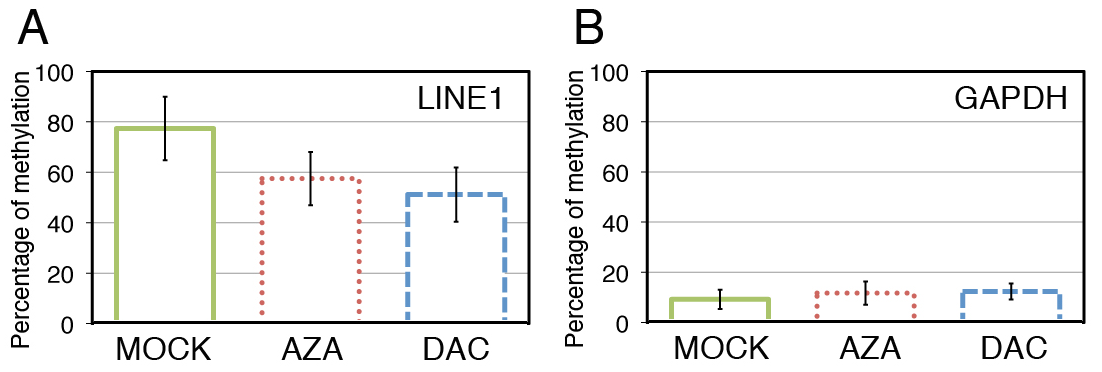

Supplement: Figure S3 — A-B, Pyrosequencing of LINE1 and GAPDH for mock- and drug-treated cells. Percentage of methylation was calculated as the mean (± SEM) at 4 (LINE1) and 5 CpG sites (GAPDH) in four independent experiments. (TIF) [file pone.0071099.s003.tif]

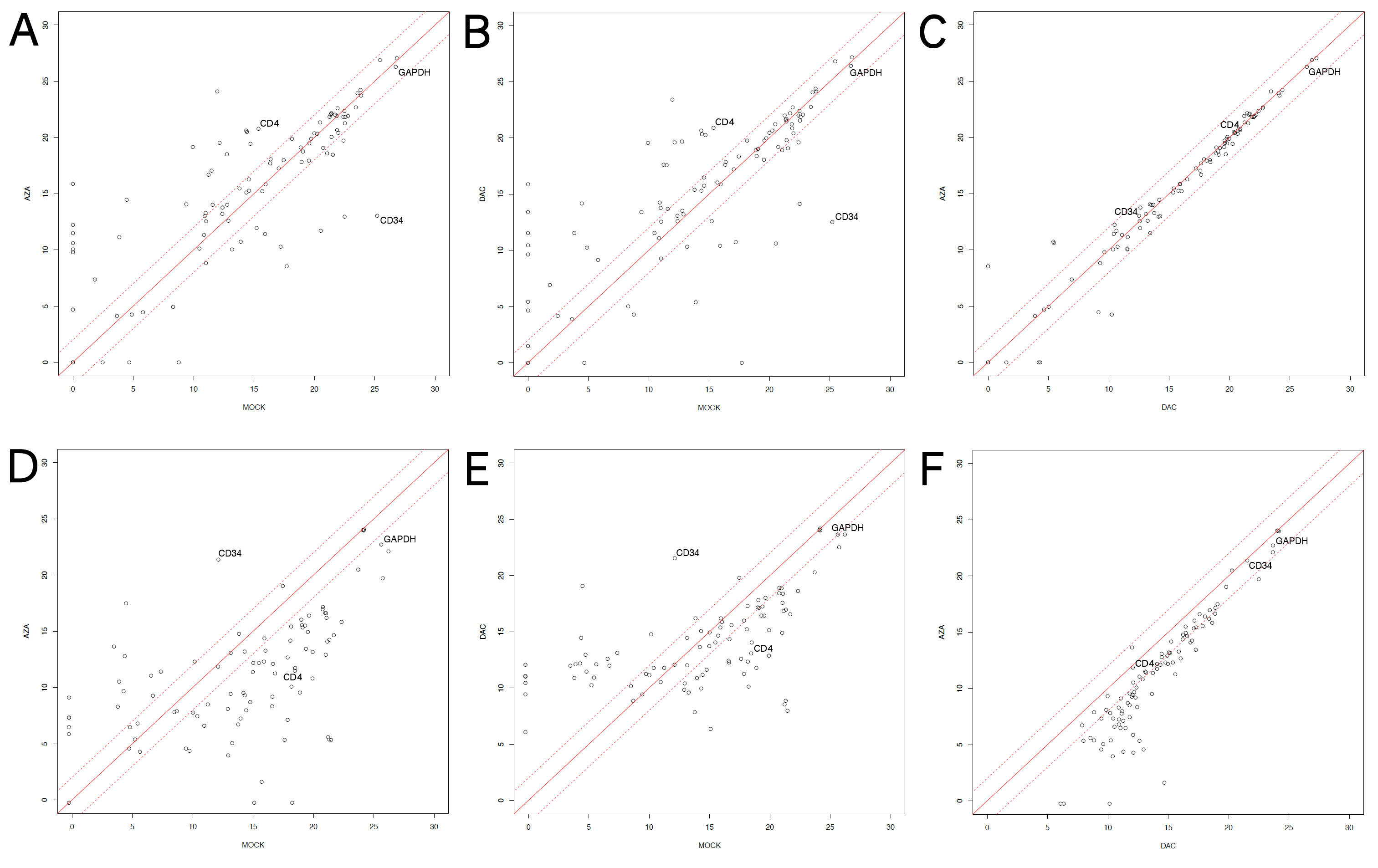

Supplement: Figure S4 — A-C, low-dose (100nM AZA, 10nM DAC) treatment for 28 days. D–F, high-dose (10mM AZA, 1mM DAC) treatment for 3 days. Relative expression is shown as M-Ct where M was defined as 3 more than the maximum Ct value obtained. Ct values for undetected genes were set to M to allow visualisation. (TIF) [file pone.0071099.s004.tif]

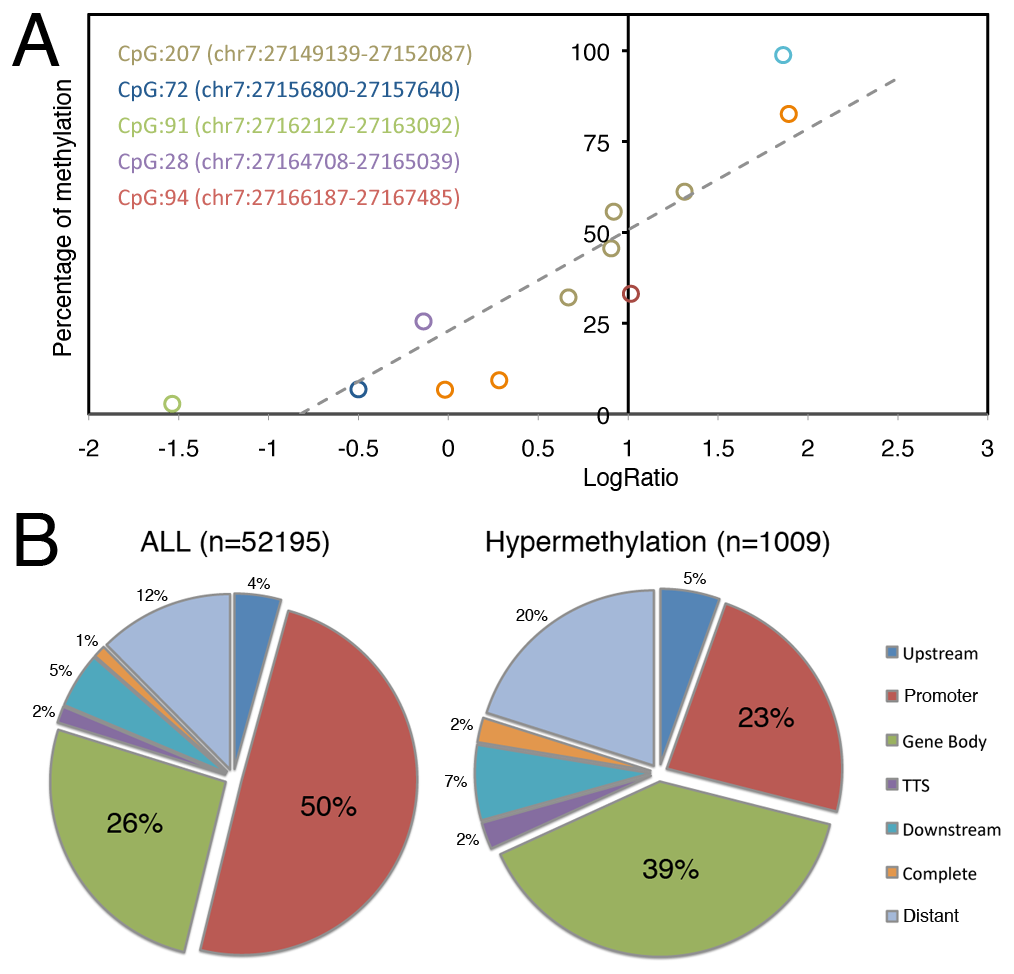

Supplement: Figure S5 — A, log2-ratios from microarray data were plotted against average methylation value from bisulfite sequencing at 15 genomic regions in the HOXA cluster. The trend line intersects 50% of methylation at log2-ratios of 1.0. B, Percentages of probes (from a subset of 52195 probes) from different island classes as defined by their location with respect to gene features (left). Hypermethylated probes (log2-ratios > 1.5) were under-represented in promoters (p<6E-66) and overrepresented in gene bodies (p<1E-20) (right). (TIF) [file pone.0071099.s005.tif]

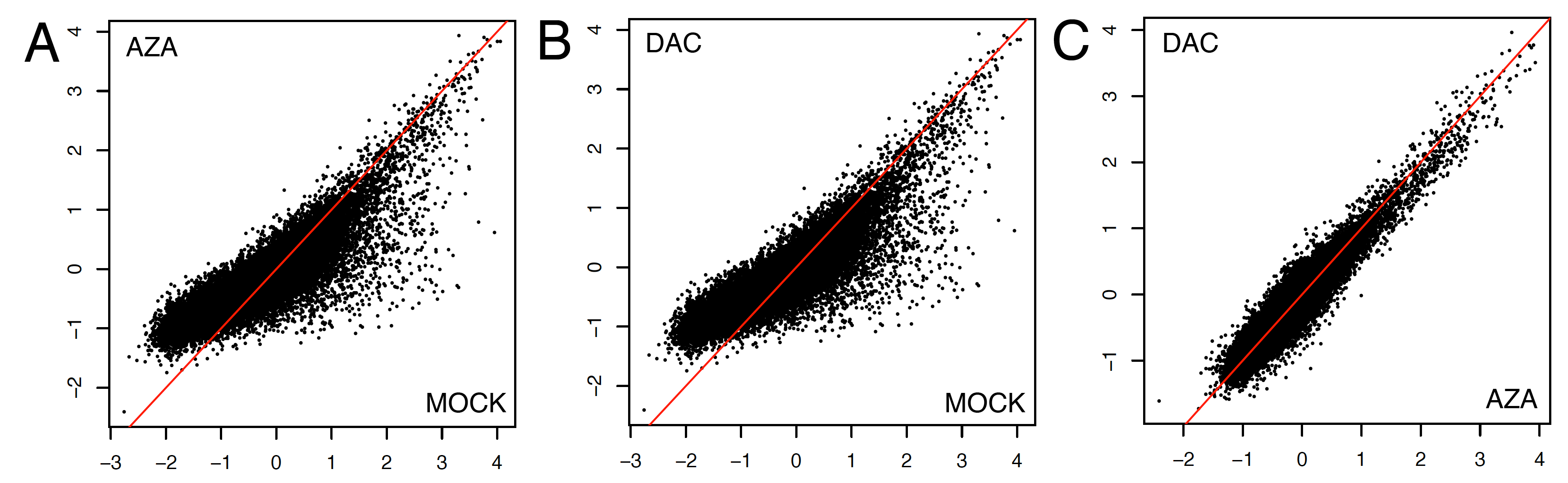

Supplement: Figure S6 — Probe log2-ratios from AZA (A) and DAC (B) treated samples were plotted against MOCK treatment log2-ratios. (C) AZA and DAC log2-ratios plotted against each other indicating an equivalent effect. (TIF) [file pone.0071099.s006.tif]

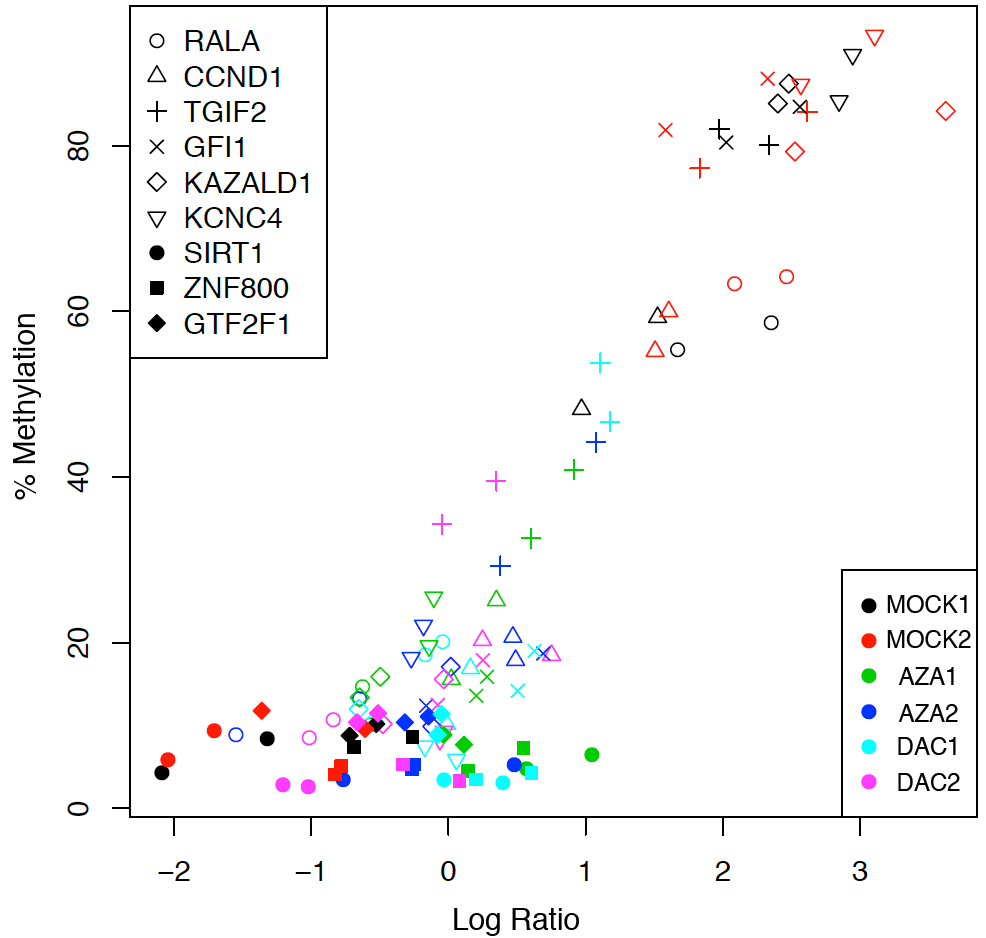

Supplement: Figure S7 — Bisulfite sequencing was performed for all sample series for MOCK, AZA, and DAC treated samples for genomic regions showing either increases or decreases in methylation after treatment. The percentage of methylation determined by bisulfite sequencing was plotted against log2-ratios from microarray data. Log2-ratios show a clear correlation with percent methylation for regions with more than 10% of CG positions methylated, but show no clear correlation for lower levels of methylation. (TIF) [file pone.0071099.s007.tif]

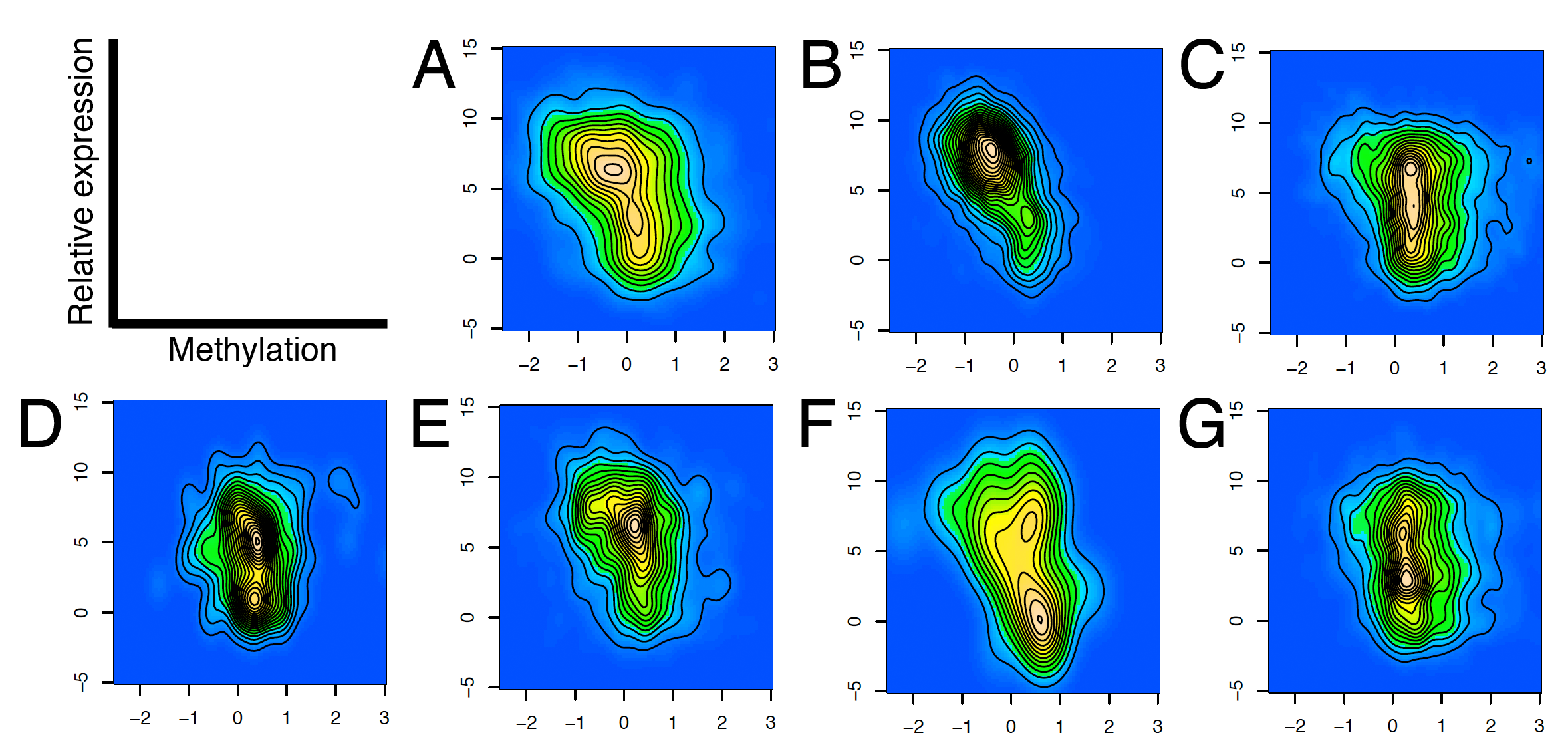

Supplement: Figure S8 — Expression in mock treated samples (log2 transformed Mas5 values) derived from Affymetrix arrays were plotted against CGI methylation (mean log2-ratios for probes from individual islands) for probes lying in CGIs associated with (A) Upstream, (B) promoter, (C) gene body, (D) TTS, (E) downstream, (F) complete, (G) distant regions (see materials and methods for definitions). (TIF) [file pone.0071099.s008.tif]

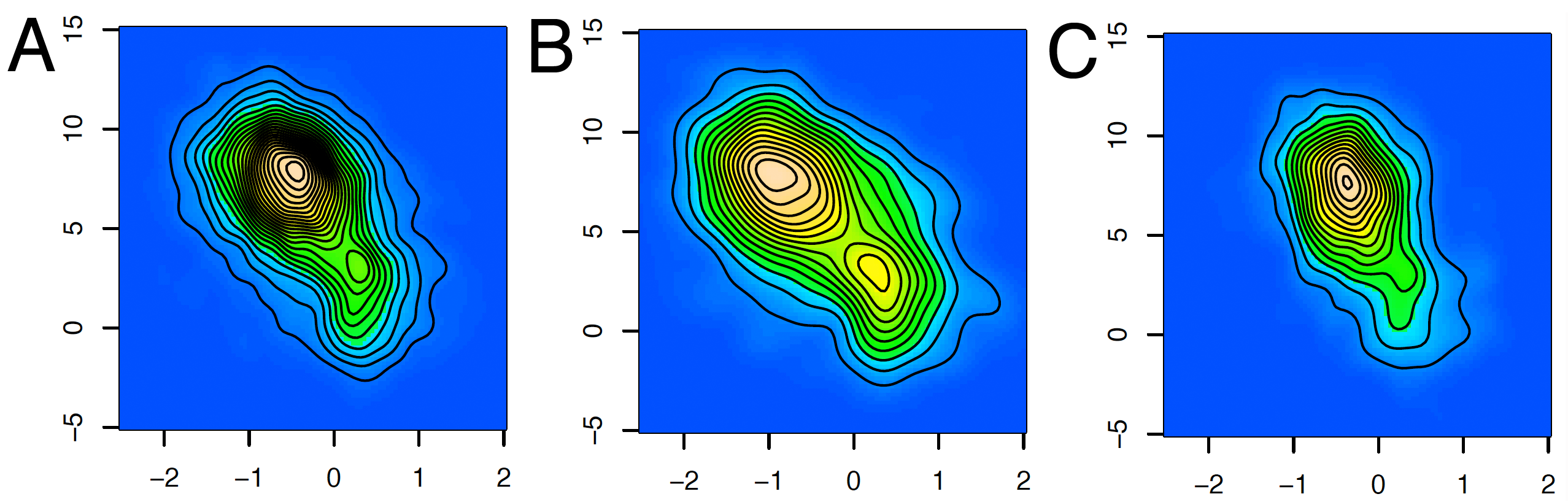

Supplement: Figure S9 — For mock-treated SKM-1 cells, expression (as in S8 & S9) was plotted against CGI methylation for all probes associated with promoter CGIs (A), and for those associated with the 5th lowest (B) and highest (C) CG density promoter associated islands. (TIF) [file pone.0071099.s009.tif]

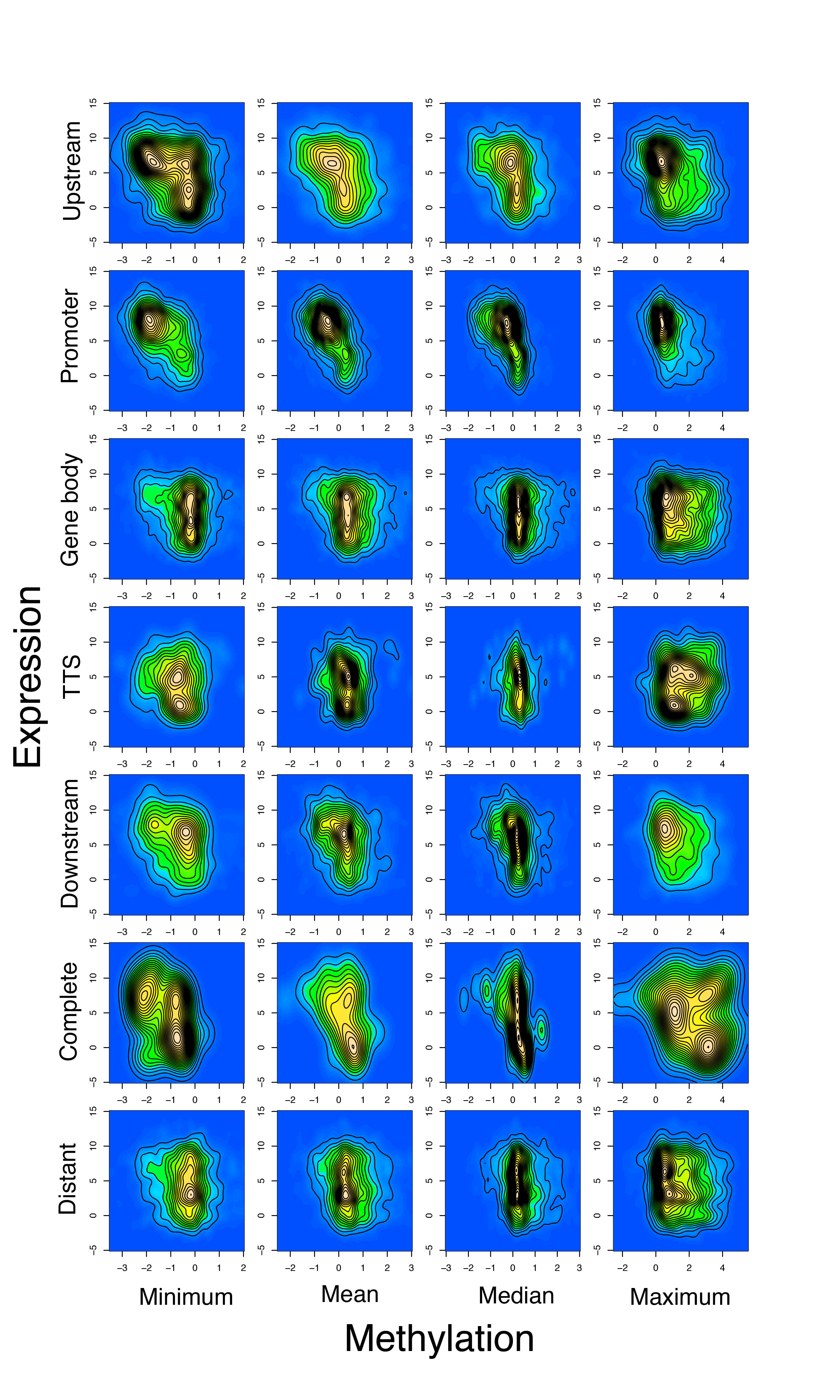

Supplement: Figure S10 — Expression was plotted against several CGI methylation summary statistics for the different classes of CGI as in Figure S8. CGI methylation was summarised by the minimum, mean, median or maximum probe log-ratio. The use of the minimum log-ratio suggests a relationship between gene expression at non-promoter islands (upstream, gene body, downstream and complete), but this does not appear to be a general relationship and may be true for a subset of the probes only. (TIF) [file pone.0071099.s010.tif]

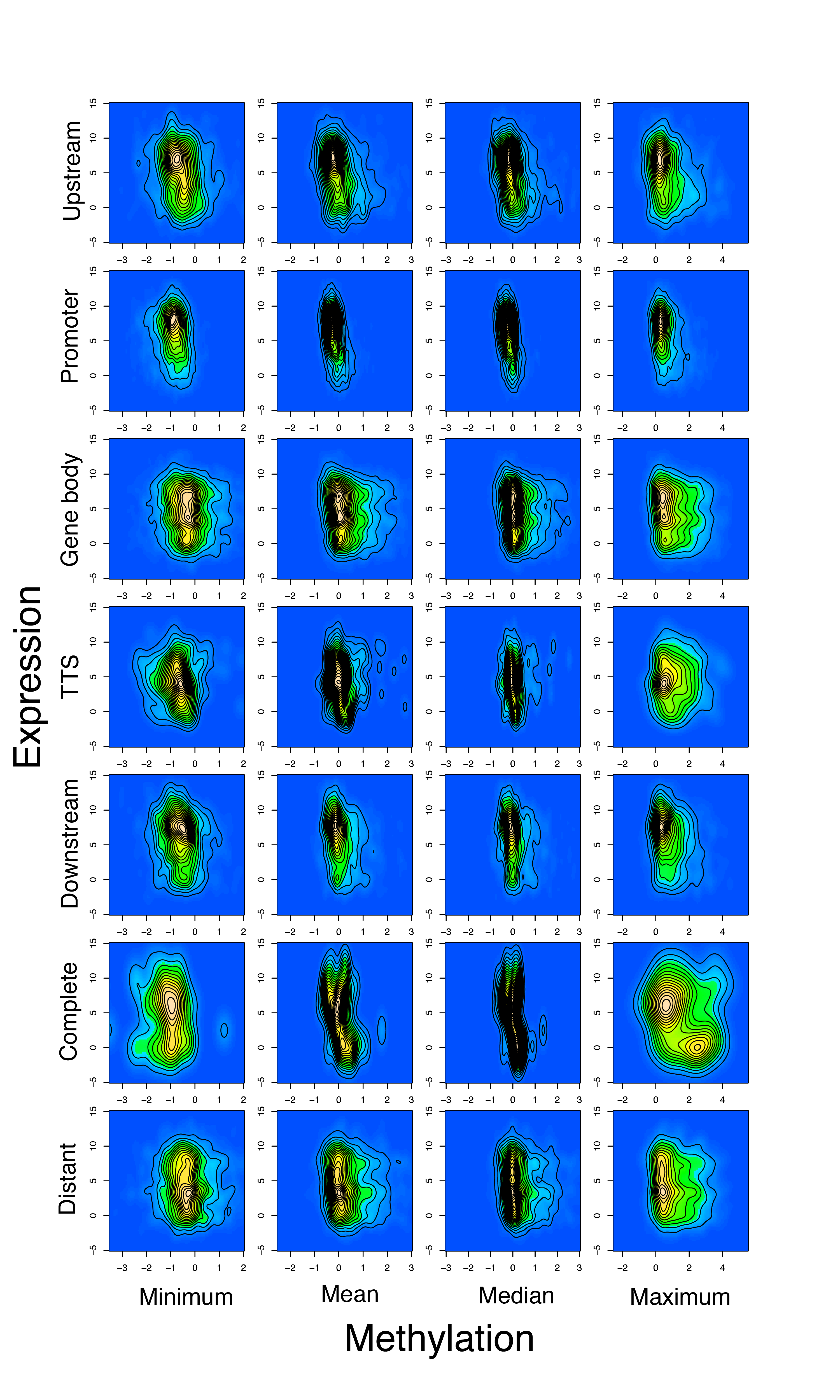

Supplement: Figure S11 — Plots as in S10 for an AZA treated sample. The relationships between methylation and expression observed in control samples are no longer present. (TIF) [file pone.0071099.s011.tif]

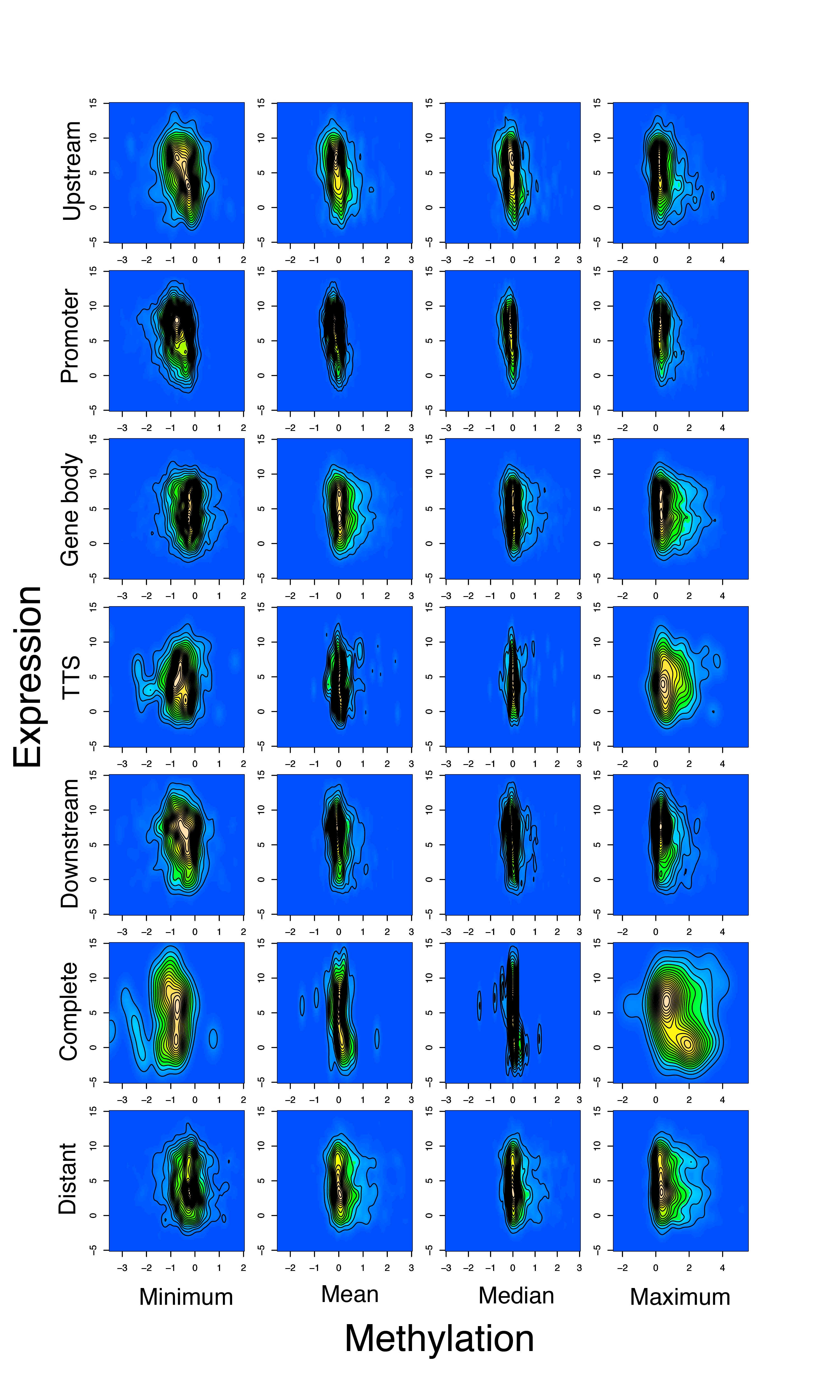

Supplement: Figure S12 — Plots as in S11 for a DAC treated sample. (TIF) [file pone.0071099.s012.tif]

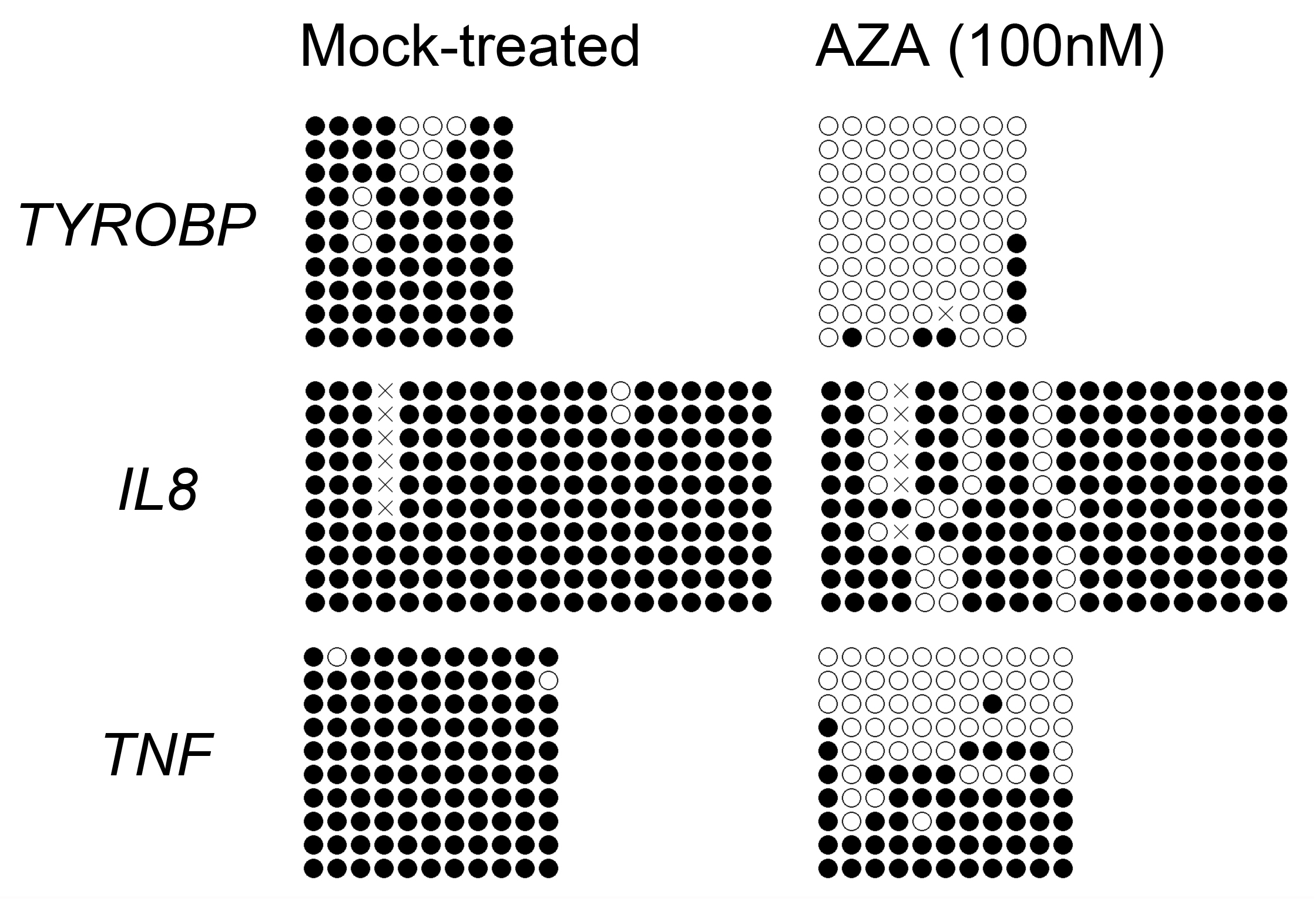

Supplement: Figure S13 — The extent of CpG methylation was determined for target sequences within FOXD1, IL8, TNF, and TYROBP by bisulfite sequencing for DNA derived from mock and prolonged AZA treated SKM-1 cells. Bisulphite sequence data from 10 independent clones are shown. Black circles represent methylated CpG sites. Dots marked with a cross were not analyzable by sequencing. (TIF) [file pone.0071099.s013.tif]
