# Supplementary material for: Prolonged Treatment with DNMT Inhibitors Induces Distinct Effects in Promoters and Gene-Bodies
Source: PLoS One. 2013 Aug 6;8(8):e71099. doi: 10.1371/journal.pone.0071099 (PMC3735498; doi:10.1371/journal.pone.0071099)
Supplement: Table S1 — (PDF) [file pone.0071099.s014.pdf]

Primers for bisulfite sequencing

BSPCR regions	Forward primers	Reverse primers	Probes
HOXA region 1	TTTTTTTGTTGTTGATGTGGGT	AAACACACATATCAAAAAACAAATAAA	A_17_P15920140
HOXA region 2	GTATGAGGGATAGTGTTTTTGT	CACTACCAAAAAATACTTCCAT	A_17_P15920148
HOXA region 3	TTTGGAAGTTAGGATTTTGG	CATCAAATCATCTCTAACCAATAAA	A_17_P15920151
HOXA region 4	TTTGGGTATTTTTGTGGA	CAAATCAAAATCTAATTCCAAA	A_17_P05377911
HOXA region 5	TGTAATTAGTTTTTATGGTGTTGG	TATTTCCCCAACTTAAAACAAT	A_17_P15920162, A_17_P15920163
HOXA region 6	TGTTTTAAGTTGGGGAAATAAT	ACTAATTCAACCAAAATTCTCC	A_17_P15920165
HOXA region 7	TTGGGTTATAAAAAGTTTTATGAGTTT	TACTCCCAACTCACAAAAAAAC	A_17_P05378001
HOXA region 8	TTTTTTTGTGAGTTGGGAGTAA	CCAAACTTAATCCAAAACTCACT	A_17_P05378009
HOXA region 9	TTAATAAATTTTTTTTGGGGTG	AAATCAAACCACAAAAAACAA	A_17_P15920183
HOXA region 10	GTGTGAAAGGAAAGAGAGAATAAATAT	AAACCAAAAACTACTTTAATTATTTTT	A_17_P05378034, A_17_P15920184
HOXA region 11	TGGTTTGTTTTTTAGGATTTT	CCCCAAATTTTAATAAACAACC	A_17_P15920185, A_17_P15920186
HOXA region 12	GTTGTTTATTAAAATTTGGGG	CAAACAAAAAAAACTTCAAAA	A_17_P15920189
GFI1	TTGGAGATTTTATAGGGAAGGG	ACTATCAATTTAAACCCAAAAACCC	A_17_P15085666, A_17_P15085667, A_17_P00378159, A_17_P00378160
KCNC4	TTTTTAGAGGTAGGTATTGTGGGA	TAACCCAAAACACCCATAATCTT	A_17_P00452908, A_17_P00452909
RALA	GGTATTTAATTTGTGGGGAGTAAAG	CTTCTAAAAAATCAACTCATATTCCC	A_17_P05435627, A_17_P05435628, A_17_P05435629, A_17_P15932310
KAZALD1	AGGAGTTGTTTTTTGAATTTAAGTT	TAAACATCCTTACCCAAAAAAA	A_17_P07431820, A_17_P07431821
CCND1	TAGGGTTATTTTTTTGGGTATT	CCAATTTTCATAAAAATACAAA	A_17_P07858632, A_17_P07858633, A_17_P07858634
TGIF2	TATGTGTTTTTTGGGTTTAGGT	CTTCTACAAATCCTAACTTTTATCA	A_17_P11149286, A_17_P17189849
ZNF800	GGAGGTAGTTGATAGGAGGAAT	AAACTTATCACTTCCTACCCTC	A_17_P16015102, A_17_P05768474, A_17_P05768475
SIRT1	GGTTTTTATTGGTTTGAGGTTG	ACCAAAAAAAACAAACTACACAAA	A_17_P07291649, A_17_P07291650
GTF2F1	TGGTTTTTTATGGGTTTGTTT	AAATCCTACATCTCCCTCCTATT	A_17_P17102993, A_17_P17102994, A_17_P17102995
TYROBP	ATAGGGGAGGAGAGATAGAAAT	ATAAAACCCTATCTCTACTAAAAAAAA	
IL8	TTAGTTTTATGGAGTTTGATGGAA	AAATCTATCACCCAAAAAAAAAAA	
TNF	TTGGTTTTTAAAAGAAATGGAG	ACCAACAACTACCTTTATATATCCC	

Primers for pyrosequencing	Forward primers	Reverse primers	Sequencing primers
HSPA2	GTAGGAAGGGTTTAAATGTAGGT	bio-AAATCACCCCACTACACTCCAACCT	GGGTTTTTTTTTGTTTTTTTTGA
TNF	AGGGGTATTTTTGATGTTTGTGT	bio-CCTTAATAAAAAAACCCATAAACTCATCTA	TTTGTGTGTTTTTAATTTTTTAAAT
TYROBP	AGATATTTAGGGGTTATGTTATAGGT	bio-AATCATAACACTAACCCTCCTATT	GGTTATGTTATAGGTTTTTAGTAAT

Primers for Real Time PCR	Forward primers	Reverse primers
HSPA2	GCACCGGTAAGGAAAACAAA	ATAGGACTCCAGGGCGTTTT
TNF	TGCTTGTTCCTCAGCCTCTT	TGGGCTACAGGCTTGTCACT
TYROBP	TGGTGCTGACAGTGCTCATTGC	CTGATAAGGCGACTCGGTCTCA
