# Supplementary material for: Prolonged Treatment with DNMT Inhibitors Induces Distinct Effects in Promoters and Gene-Bodies
Source: PLoS One. 2013 Aug 6;8(8):e71099. doi: 10.1371/journal.pone.0071099 (PMC3735498; doi:10.1371/journal.pone.0071099)
Supplement: Table S3 — The numbers of probes from each class was calculated for all (top row) or for probes with a maximal log2 within-group variance below the indicated thresholds (as used to select probes for further analysis in the main section). (PDF) [file pone.0071099.s016.pdf]

threshold	upstream	gene body	promoter	downstream	TTS	Complete	Other
	8247	57494	91991	9847	2744	2271	25708
2	8210	57009	91669	9796	2725	2249	25543
0	7072	45782	80829	8341	2264	1852	21033
-2	3199	19359	37717	3830	1083	818	9176
-4	535	3764	6289	681	256	174	1771
