# Supplementary material for: Prolonged Treatment with DNMT Inhibitors Induces Distinct Effects in Promoters and Gene-Bodies
Source: PLoS One. 2013 Aug 6;8(8):e71099. doi: 10.1371/journal.pone.0071099 (PMC3735498; doi:10.1371/journal.pone.0071099)
Supplement: Table S4 — The proportions of probes from each class were calculated for all (top row) or for probes with a maximal log2 within-group variance below the indicated thresholds. No change in proportion is seen as probes are selected by increasing stringency. (PDF) [file pone.0071099.s017.pdf]

threshold	upstream	gene body	promoter	downstream	TTS	Complete	Other
	0.04	0.29	0.46	0.05	0.01	0.01	0.13
2	0.04	0.29	0.46	0.05	0.01	0.01	0.13
0	0.04	0.27	0.48	0.05	0.01	0.01	0.13
-2	0.04	0.26	0.50	0.05	0.01	0.01	0.12
-4	0.04	0.28	0.47	0.05	0.02	0.01	0.13
