# Supplementary material for: Prolonged Treatment with DNMT Inhibitors Induces Distinct Effects in Promoters and Gene-Bodies
Source: PLoS One. 2013 Aug 6;8(8):e71099. doi: 10.1371/journal.pone.0071099 (PMC3735498; doi:10.1371/journal.pone.0071099)
Supplement: Table S5 — Probes demethylated as a result of AZA or DAC treatment were determined as described in the main text. These were considered as having been sampled from the set of probes hyper-methylated in the control samples. The probability of observing at least or at most the number of probes identified from each individual class was calculated using Fisher’s exact test for over- and under-representation respectively. (PDF [file pone.0071099.s018.pdf]

	upstream	gene body	promoter	downstream	TTS	Complete	Other
	over-represented						
AZA	0.010	1	1.6E-14	0.001	0.026	0.363	0.965
DAC	0.011	1	6.4E-16	0.002	0.048	0.251	0.991
Common	0.011	1	1.5E-14	0.002	0.041	0.322	0.954
	under-represented						
AZA	0.994	2.0E-20	1	0.999	0.988	0.743	0.045
DAC	0.994	9.2E-20	1	0.999	0.976	0.834	0.012
Common	0.994	2.0E-20	1	0.999	0.980	0.778	0.058
